# But What’s Your Partner Up to? Associations Between Relationship Quality and Pornography Use Depend on Contextual Patterns of Use Within the Couple

**DOI:** 10.3389/fpsyg.2021.661347

**Published:** 2021-07-30

**Authors:** Taylor Kohut, Kiersten A. Dobson, Rhonda N. Balzarini, Ronald D. Rogge, Amanda M. Shaw, James K. McNulty, V. Michelle Russell, William A. Fisher, Lorne Campbell

**Affiliations:** ^1^Department of Psychology, Western University, London, ON, Canada; ^2^Department of Psychology, University of Rochester, Rochester, NY, United States; ^3^Department of Psychology, University of Florida, Tallahassee, FL, United States

**Keywords:** pornography, romantic relationship, relationship satisfaction, sexual satisfaction, erotica

## Abstract

It is commonly assumed that exposure to pornography harms relationships because pornography changes the way that individuals think, feel, and behave in problematic ways. In the current research, we contribute to a small but growing body of work that challenges this assumption by carefully scrutinizing the relational context of pornography use. In contrast to dominant theoretical explanations in this field, we argue that at least some of the apparent negative “impacts” of pornography use on relationship quality may reflect partner dissimilarity in pornography use behavior rather than the consequences of exposure to such materials. Moreover, we further examine a particular type of pornography use – shared use with a partner – which previous evidence suggests may be positively associated with relationship quality. To this end, we sought to test whether dyadic patterns of pornography use, and related attributes, were associated with sexual and relationship satisfaction in two cross-sectional (*N*_1_ = 200; *N*_3_ = 207) and two longitudinal (*N*_2_ = 77; *N*_4_ = 277) samples of heterosexual couples. Across these samples, we found consistent evidence that partners who watch pornography together report higher relationship and sexual satisfaction than partners who do not, and notably, this association was not moderated by gender. Independent of this association, we also found evidence of a similarity-dissimilarity effect, such that the solitary pornography use of one partner was negatively associated with their own relationship and sexual satisfaction, but only in cases where their romantic partners used little or no pornography alone. Further consideration of several correlates of pornography use established comparable patterns of results for dissimilarity in attitudes toward pornography, erotophobia-erotophilia, sexual preferences, and sex drive. Importantly, only dissimilarity in sex drive statistically accounted for dissimilarity in solitary pornography use, suggesting that differences in sex drive may be implicated in the associations between pornography use and relationship quality. These findings demonstrate that links between pornography use and relationship health are partially a function of different dyadic patterns of pornography use within couples and do not always suggest relational harm.

## Introduction

Media (Montgomery-Graham et al., 2015) and academic (Zillmann, 2000; Manning, 2006) claims that pornography use undermines romantic relationships are widespread. However, recent failed replications (Balzarini et al., 2017), conceptual and empirical critiques (Campbell and Kohut, 2017; Perry, 2019; Fisher and Kohut, 2020; Kohut et al., 2020), and opposing findings (Kohut et al., 2018) are increasingly challenging this view. In the current research, we focus on different patterns of pornography use within adult relationships in an effort to reconcile evidence that pornography use may be related to both enhanced and diminished relationship functioning.

### Pornography Use and Relationship Functioning

Although pornography use is a simple behavior to engage in, it has proven to be a difficult concept to define. While many definitions have been proposed, we have adopted the empirically informed conceptual definition described by Kohut et al. (2020) for the current paper because it attempts to embed the understanding of this construct within a complex network of potential antecedent-consequent relationships. At the heart of this theoretical definition, pornography use is understood to be a “common but stigmatized behavior, in which one or more people intentionally expose themselves to representations of nudity which may or may not include depictions of sexual behavior” (Kohut et al., 2020, 732).

Several theories have been advanced to explain pornography’s impacts on relationships (e.g., _3_AM, Social Learning Theory, Social Comparison Theory, the Confluence Model, the Elaboration Likelihood Model/Heuristic-Systematic Theories, etc.). At its core, Wright et al. (2014) _3_AM, for example, argues that viewing pornography will contribute to the acquisition of new sexual scripts, the activation of previously acquired scripts, and/or the application of such scripts with respect to decisions about one’s own behavior or judgments about the behavior of others. Other perspectives, such as those that apply Social Comparison theory (Festinger, 1954) or similar theories to the issue pornography (Kenrick et al., 1989; Muusses et al., 2015; Wright et al., 2019), focus more on how pornography users compare themselves and their romantic partners to what they see in pornography and change how they feel about their own sexual lives as a result. These are just two examples of many theoretical approaches to explaining pornography’s effects, but what nearly all explanations of the effects of pornography have in common is that they assume that *exposure* to the content of sexual media precipitates personal or interpersonal change (see Davis and Bauserman, 1993; Hogben and Byrne, 1998; Malamuth et al., 2000; Fisher and Barak, 2001; Wright et al., 2014; Muusses et al., 2015; Leonhardt et al., 2019). While few of these theories argue that the impacts of pornography *must* be harmful, specific applications of such theories have typically asserted that the content of pornography drives the acquisition of sexual scripts; influences sexual attitudes, standards, and expectations; or changes perceptions of personal or partner characteristics in ways that are deleterious to relationship functioning.

From this exposure-based perspective researchers have sought – and frequently found – evidence that pornography use is related to relationship dysfunction. Examples include studies that have found that pornography use is associated with decreased sexual and relationship satisfaction, particularly among men (Wright et al., 2017), impaired love for, and attraction to one’s relationship partner (Weaver et al., 1984; Zillmann and Bryant, 1988; Kenrick et al., 1989), reduced relationship commitment (Lambert et al., 2012), increased extra-dyadic sexual behavior (Yucel and Gassanov, 2010; Maddox et al., 2011; Lambert et al., 2012), and relationship dissolution (Perry and Davis, 2017; Perry and Schleifer, 2018). With this accumulated empirical evidence and the underlying exposure-based theoretical rationales that accompany it, it is tempting to conclude that pornography does indeed undermine the well-being and stability of romantic relationships.

There remain, however, several reasons to be cautious about strong conclusions regarding pornography’s negative impacts on relationships. For example, there is ongoing concern across the social sciences that many, perhaps most, of our previously accepted scientific findings are not reliable (Spellman, 2015). Close replications of research in this specific area are rare, but the only published attempt that we are aware of has not corroborated evidence that exposure to sexual images reduces love for or attraction to men’s romantic partners (Balzarini et al., 2017), and such findings confirm older, rarely cited, conceptual replications that have not found such effects either (Dermer and Pyszczynski, 1978; Amelang and Pielke, 1992).

There is also a clear disparity between the conclusions reached by studies that examine the relationship correlates of pornography use and studies that ask pornography users – and importantly, the romantic partners of pornography users – about their perceptions of the effects of pornography. The former tend to find negative correlations between pornography use and relationship functioning, as outlined above, but most individuals who are in relationships in which pornography is used do not believe that pornography has harmed their relationships (Bridges et al., 2003; Hald and Malamuth, 2008; Grov et al., 2011; Rissel et al., 2016; Kohut et al., 2017b). While some have suggested motivated reasoning may lead pornography users to under-report the harms of their use (Hald and Malamuth, 2008; Vandenbosch, 2019) available evidence for this view is sparse, and some lines of inquiry actually indicate influence in the opposite direction. It appears, for example, that negative attitudes toward pornography among pornography users may be biasing their perceptions of their use toward rather than away from harm (Grubbs et al., 2019). There is clearly much to learn here, but at present, the lack of correspondence between these two literatures should raise important questions and suggests to us that pornography’s impact on relationship functioning may be more complex than it seems.

Finally, conceptual and methodological critiques of this literature emphasize the need to be cautions when making inferences about the effects of pornography on relationship quality (Campbell and Kohut, 2017; Fisher and Kohut, 2020; Kohut et al., 2020). Such concerns run the gamut from the evident harm bias in field, to the much less discussed effects-bias among many researchers (for an apparent defense of the view that correlates of pornography frequently represent causal effects, see Wright, 2021a, b), to more general problems with sampling, measurement, and generalizability across research. Most critical for this discussion is the concern expressed by Kohut et al. (2020) that researchers do not always fully understand and reflect on the complex nature of pornography use, and as a consequence, risk making inferential errors about pornography’s presumed effects.

### Antecedents, Context, and Effects of Pornography Use

In an effort to explain some of the discrepant and divergent findings, Campbell and Kohut (2017) have encouraged researchers to adopt an Antecedents-Context-Effects (ACE) perspective in this field of research. According to the ACE approach, the causal effects of pornography use may vary as a function of different *contexts* of pornography use within relationships (e.g., solitary vs. shared use, hidden vs. open use, the consumption of pro- vs. anti-social content, etc.), which in turn may indicate unique *antecedents* of use (e.g., sex drive, erotophobia-erotophilia, sexual attitudes, etc.^1^; see also Leonhardt et al., 2019). Suppose, for example, that a relationship is characterized by large discrepancies in erotophobia-erotophilia. Erotophilic individuals are theorized to have had a lifetime of socialization experiences that reinforce a tendency to approach sexual cues in their environment and respond to such cues with positive affect (Fisher et al., 1988). In contrast, erotophobic individuals are expected to have been socialized to avoid such cues, and respond to such cues with negative affect. An erotophilic individual in such a couple would be more likely to be drawn to pornography use, would likely keep their use hidden from the more erotophobic partner who might respond to knowledge of such use with anger and disgust, and will be unlikely to use pornography with their partner. The relationship sequelae of this pattern of pornography use are likely quite different from cases where both partners are erotophilic, as such couples may be more open and honest about their pornography use, and may be more likely to use it together in addition to their solitary use.

#### Solitary vs. Shared Pornography Use

One prominent division that has been proposed with respect to different relationship *contexts* of pornography use is the distinction between solitary and shared pornography use (Campbell and Kohut, 2017). In general, studies have found that relationship quality is positively correlated with the extent that relationship partners use pornography together (Kohut et al., 2018; Huntington et al., 2020; Willoughby and Leonhardt, 2020). Other research designs also suggest that relationship quality is higher among individuals who use pornography together with a romantic partner compared to individuals who only use pornography alone (Bridges and Morokoff, 2011; Maddox et al., 2011).

Explanations for such effects have been sparse and poorly developed, but one possibility, echoed by both the perceptions of pornography users themselves (Kohut et al., 2017b), as well as clinicians (Manning, 2006), is that shared pornography use provides opportunities to learn about each other’s sexual likes and dislikes and build intimacy together (Kohut et al., 2018). This process can be understood from theoretical perspectives that emphasize the importance of the shared nature of novel and exciting activities which provide opportunities for “self-expansion” (Aron et al., 2000) and personal self-disclosures (Reis and Shaver, 1988). Exposure-based theories of pornography’s impact may also play a role, in that joint exposure to the same sexual media may enhance the similarity of partners’ sexual attitudes, scripts, and expectations over time. In particular, Leonhardt et al. (2019) have suggested that exposure to scripts in sexual media should reinforce the pursuit of physical sexual pleasure and variety at the expense of affection, attachment, and commitment toward one’s partner. Others believe that this position mischaracterizes the nature of sexual scripts in pornography (Kohut and Campbell, 2019), pointing out that even the depiction BDSM practices – which Leonhardt et al. (2019) include in their description of the most detrimental materials for relationships – exhibit relationship enhancing scripts.

#### Similarity and Dissimilarity

Until recently, research in this area has frequently failed to consider dyadic patterns of solitary pornography use across both partners (Campbell and Kohut, 2017). What is often overlooked is that within a relationship involving two people, partners can be similar in their extent of solitary pornography use or non-use, or they can be dissimilar in their solitary pornography use, as when one partner uses pornography alone very frequently and the other does not. Although dyadic research which collects data from both relationship partners is beginning to accumulate, most studies still do not consider how partner similarity-dissimilarity in solitary pornography use is related to relationship functioning. Among studies that have, evidence suggests that pornography use tends to be more strongly associated with sexual and relationship dysfunction when one partner uses it and the other does not (Daneback et al., 2009; Yucel and Gassanov, 2010; Willoughby et al., 2016; Kohut et al., 2018).

The possibility that similarity-dissimilarity in solitary pornography use may be relevant to relationship quality should not be surprising. It has long been known in the study of close relationships that similarity in attitudes (Byrne, 1971; Montoya and Horton, 2013), personality, demographic variables (Buss, 1985), and recreational interests (Werner and Parmalee, 1978; Boer et al., 2011) promotes interpersonal liking. Within the specific context of romantic relationships, research also suggests that similarity in sexual preferences (Purnine and Carey, 1999), sexual ideals (Balzarini et al., 2021), erotophobia-erotophilia (Fisher et al., 1988; Smith et al., 1993), and sex drive (Eysenck and Wakefield, 1981) are all related to increased attraction and/or relationship functioning. With respect to such effects, it has long been believed that similar others validate our worldview and that this validation facilitates liking through positive reinforcement (Byrne, 1971). Emerging research, however, favors an alternative information-processing perspective where traits that are similar to one’s own are viewed positively in and of themselves, and this is what drives attraction (Montoya and Horton, 2013). According to this perspective, traits that are dissimilar from one’s own are viewed less positively, or even negatively, which leads to disliking.

On the basis of this literature, similarity and dissimilarity in dyadic patterns of solitary pornography use within relationships should also be differentially related to relationship functioning even if exposure to pornography fails to influence people thoughts, feelings, and behaviors. It could be, for example, that the discrepancy in pornography use between partners is a direct source of conflict in some relationships. Non-using individuals, if they are aware of their partners’ pornography use, may have difficulty understanding it, view the behavior as infidelity, feel “replaced” as a romantic partner, or have self-doubts or personal insecurities as a result (Clark and Wiederman, 2000; Bergner and Bridges, 2002; Kohut et al., 2017b). Similarly, if the pornography user expects a negative response from their non-using partners, they may hide their use and lie about it to their partners (Resch and Alderson, 2014; Kohut et al., 2017b) which would likely place boundaries on intimate self-disclosures and interpersonal closeness in the relationship (Kohut et al., 2018). Under such conditions, it is likely that dissimilarity in pornography use, and not just exposure to such materials, may be undermining relationships.

Considering the ACE model, it is also possible that similarities or dissimilarities in sexual attitudes, sexual preferences, erotophobia-erotophilia, and sex drive – all of which are correlated with pornography use (Fisher et al., 1988; Davis and Bauserman, 1993; Baer et al., 2015) – may drive different patterns of pornography use within relationships. From this perspective, relationship conflicts over dissimilar pornography use may be proximal behavioral manifestations that mediate connections between broader similarity-dissimilarity in sexual attitudes (or other characteristics) and relationship functioning. Alternatively, however, Campbell and Kohut (2017) also warn that the antecedents of pornography use themselves may be the true proximal causes of the *assumed effects* of pornography use on relationship functioning. If this is true, similarity-dissimilarity in couple members’ pornography use may simply act as an observable marker of more fundamental differences between partners (for an explicit denouncement of this view, see Wright, 2021a). Lastly it is also possible that similarity-dissimilarity in sexual attitudes, erotophobia-erotophilia, and/or sex-drive may be the result of differences in pornography use (Wright, 2021a, b). Regardless of the causal mechanism that is at play, there are evident reasons to consider whether similarity-dissimilarity in factors like sexual attitudes, sexual preferences, erotophobia-erotophilia, and sex drive may be implicated in the expected associations between similarity-dissimilarity in solitary pornography use and relationship functioning.

### Gender

It is very well established in pornography research that men are more likely to report using pornography and do so at a higher frequency than women (Petersen and Hyde, 2010; Carroll et al., 2017). Furthermore, Wright et al. (2017) meta-analysis found that while men’s use of pornography is generally associated with lower sexual and relationship satisfaction, women’s pornography use is not. Such findings have reinforced speculations about gender-specific processing of pornographic content (see, for example, Wright et al., 2017).

There is, however, another possibility. Because of gender differences in base rates of pornography use, within heterosexual relationships, the probability that male pornography users are in relationships in which both people use pornography is not the same as the probability that female pornography users are in relationships in which both people use pornography. Indeed, when Kohut et al. (2018) checked their data they found that while over 80% of the female pornography users in their sample were in relationships where both partner’s used pornography alone, only half of the male pornography users were in such relationships. While this is just one sample, a general difference in conditional probabilities of this magnitude would suggest that on average, female solitary pornography use is more likely to occur within relationships in which both members use pornography than male solitary pornography use. If this is the case then gender differences in the correlates of pornography use might reflect the consequences of similarity-dissimilarity in addition to or instead of any gender-specific processing of pornography that may occur.

### Research Overview

In summary, we propose that divergent patterns of association between pornography use and relationship quality are partially a function of different dyadic patterns of pornography use within adult romantic relationships. Specifically, we argue that similarity-dissimilarity in couple members’ solitary pornography use as well as shared pornography use differentiates positive from negative relationship functioning. One question that remains is whether associations between relationship quality and similarity-dissimilarity in solitary pornography use exist independently of associations between relationship quality and shared pornography use. Although it is clear that men are more likely to use pornography than women (Petersen and Hyde, 2010; Carroll et al., 2017), and that shared pornography use is less frequent than solitary pornography use (Kohut et al., 2018; Willoughby and Leonhardt, 2020) less is known about the co-occurrence of similarity in solitary pornography use and shared pornography use within relationships. In one of the few studies to describe basic dyadic patterns of pornography use it appears that shared pornography use is particularly common within relationships in which both partners use pornography individually (Kohut et al., 2017a). Under such conditions, associations between similarity-dissimilarity in solitary pornography use and relationship functioning might partially, or even wholly, reflect associations between shared pornography use and relationship functioning (or vice versa). Although several studies have indicated that relationship quality is higher when partners are more similar rather than dissimilar in their pornography use (Daneback et al., 2009; Yucel and Gassanov, 2010; Willoughby et al., 2016; Kohut et al., 2018), and when partner’s report more shared pornography use (Kohut et al., 2018; Huntington et al., 2020; Willoughby and Leonhardt, 2020), only one study to our knowledge has examined such associations in the same statistical model. In this case, Kohut and colleagues (Kohut et al., 2018) found that such associations were independent of one another, but their examination was limited to correlations with sexual communication and interpersonal closeness. Whether these associations remain independent when correlations with other measures of relationship quality are considered remain to be seen.

We further argue that pornography use may be one of many attitudinal and/or motivational dimensions on which couple dissimilarity is related to relationship dysfunction, either because it mediates the impact of other ultimate causes of relationship dysfunction, or because it stems from such causes but is only spuriously associated with relationship dysfunction, or because such variables are themselves impacted by pornography use, ultimately mediating associations between pornography use and relationship dysfunction. While it is not possible to definitively determine which causal mechanisms is at play with cross-sectional or even longitudinal designs (Fisher and Kohut, 2020), it is still worth considering whether similarity-dissimilarity in factors like attitudes toward pornography use, sexual ideal preferences, erotophobia-erotophilia, and sex drive may be statistically confounded with similarity-dissimilarity effects for solitary pornography use. Information about such confounds gleaned from correlational designs may prove relevant for understanding causal relationships between dyadic patterns of pornography use and relationship functioning in subsequent research.

What follows is a description of an inter-laboratory collaboration that sought to determine if dyadic patterns of pornography use were related to differences in sexual and relationship satisfaction within adult relationships. Across the studies presented below we expected that the frequency of shared pornography use should be positively associated with both sexual and relationship satisfaction. Further, we expected that independent of this association, partners’ reports of solitary pornography use would interact, such that couple members who were similar in their frequencies of solitary pornography use would report greater sexual and relationship satisfaction than couple members who were dissimilar in their frequencies of solitary pornography use. In so doing, we also explored, where we could, whether similarity in attitudes toward pornography use, sexual ideal preferences, erotophobia-erotophilia, and sex drive could statistically account for associations between patterns of pornography use and sexual and relationship satisfaction. In sum, this research was guided by the following hypotheses and research question:

H1: The frequency of shared pornography use should be positively correlated with (a) relationship and (b) sexual satisfaction.

H2: The frequencies of each partners’ solitary pornography use should interact positively, such that (a) relationship and (b) sexual satisfaction would be lowest when partners were most dissimilar in their solitary pornography use.

RQ1: Are interactions between partners’ reports of solitary pornography use partially or wholly confounded with interactions between partners’ attitudes toward pornography, sexual ideal preferences, erotophobia-erotophilia, or sex-drive?

Given the theoretical positions adopted in the current paper, there are no compelling reasons to expect that the anticipated similarity-dissimilarity effects of solitary pornography use or the effects of shared pornography use should be moderated by gender. Indeed, past research that has adopted a similar theoretical approach did not find evidence of such moderation when correlations between pornography use, sexual communication, and interpersonal closeness were considered (Kohut et al., 2018). However, gender is a commonly examined variable in pornography research, and at least one other study of similarity-dissimilarity in pornography use reported some gender-specific effects (Willoughby et al., 2016), so we considered possible interactions with gender in the current research as well.

The data that serve as the basis for the current analyses are drawn from four dyadic datasets of adult couples, collected by three independent laboratories. They are presented in the order in which the data became available to the first author, as this best represents the actual development of this research project. Study 1 tested H1 and H2 in a sample of *N* = 200 married heterosexual couples. Study 2 concerned a preliminary and somewhat limited examination of RQ1 by exploring whether or not similarity-dissimilarity in attitudes toward one’s own pornography use were related to relationship and sexual satisfaction in longitudinal sample of *N* = 77 newlywed couples. Study 3 replicated tests of H1 and H2 and further examined RQ1 more thoroughly by considering whether similarity-dissimilarity in sexual ideals and erotophobia-erotophilia confounded associations with similarity-dissimilarity in solitary pornography use in a cross-sectional sample of *N* = 207 heterosexual couples. Finally, Study 4 tested H1 and H2 once again, and further scrutinized RQ1 by determining whether or not associations with similarity-dissimilarity in solitary pornography use were statistically confounded with similarity-dissimilarity in attitudes toward a partners’ use of pornography and sex drive in a longitudinal sample of *N* = 277 heterosexual couples. Apart from Study 1, where there was an administrative error, we pre-registered our hypotheses and analytic plans prior to conducting our planned analyses but after the data were collected for other purposes.

## Study 1: Pornography Use and Sexual and Relationship Satisfaction

Research has previously found that comfort with sexual communication and interpersonal closeness are independently associated with both the frequency of shared pornography use and partners’ similarity in solitary pornography use (Kohut et al., 2018). Based upon such findings, it seemed likely that patterns of pornography use should also be associated with the closely related constructs of sexual and relationship satisfaction. Consequently, Kohut et al.’s (2018) data were used to test H1 and H2 and to examine RQ1 (registered materials: https://osf.io/p9ut3; data and syntax^2^). Details of related ancillary research questions concerning curvilinear associations between pornography use and relationship quality and their results can be found in Supplementary Data Sheet 1.

### Study 1: Method

#### Study 1: Participants

The sample consisted of *N* = 200 American heterosexual couples consisting of 400 individuals. These couples were quota sampled through Qualtrics Panel LLC so that women in the sample better reflected the distribution of age and political affiliation of married American women between 25 and 44 years of age. Estimates for these distributions were derived from the General Social Survey (Smith et al., 2019). Full details concerning the sampling approach and data exclusions can be found in Kohut et al. (2018). Couple members were predominately middle-aged (*M* = 41.81), Caucasian (83.5%), Christians (54.25%), with a range of political viewpoints, and were in married or common-law relationships (96.50%) of a mean duration of nearly 15 years.

#### Study 1: Materials and Procedure

After informed consent was verified in an online procedure, participants were asked to complete demographic items and established measures of relationship satisfaction, sexual satisfaction, interpersonal closeness, sexual communication, attachment orientation, and pornography use. Participants were then debriefed and provided token compensation. The local research ethics board reviewed and approved the materials and procedures before study initiation. Means and standard deviations for the following measures can be found in Table 1.

**TABLE 1:** Summary of the correlations, means, and standard deviations of the focal variables for Study 1 (*N* = 200 couples).

	**1**	**2**	**3**	**4**	**5**	**6**	**7**	**8**	***M***	***SD***
1. Relationship Satisfaction Male									0.12^1^	0.81
2. Relationship Satisfaction Female	0.65**								–0.12	0.93
3. Sexual Satisfaction Male	0.66**	0.52**							5.97^1^	1.29
4. Sexual Satisfaction Female	0.44**	0.70**	0.52**						5.70	1.50
5. Solitary Porn. Use Male	−0.22**	−0.14*	–0.10	–0.13					3.01^1^	2.01
6. Solitary Porn. Use Female	–0.08	–0.02	–0.04	0.03	0.38**				1.89	1.28
7. Shared Porn. Use Male	0.06	0.14*	0.12	0.09	0.37**	0.35**			1.77	1.19
8. Shared Porn. Use Female	0.06	0.10	0.06	0.06	0.29**	0.48**	0.76**		1.76	1.14
9. Mean Shared Porn. Use	0.06	0.13	0.10	0.08	0.35**	0.44**	0.94**	0.94**	1.77	1.10

*Signifies significant correlations *p* < 0.05; **signifies significant correlations, *p* < 0.01.

*^1^Men report significantly higher relationship satisfaction, *p* < 0.001, sexual satisfaction, *p* = 0.005, and pornography use, *p* < 0.001, than women.*

*For interpretability, all *M* and *SD* are presented in the original scale metric.*

##### Study 1: relationship satisfaction

Relationship satisfaction was measured with the four item short-form of the Couples Satisfaction Index (Funk and Rogge, 2007). Participants were asked to respond to items such as, “Please indicate the degree of happiness, all things considered, of your relationship” with 6- or 8-point scales. As a result of a programing error on the survey platform, the item “In general, how satisfied are you with your relationship?” had seven rather than the intended six response options. An additional response option “Very Satisfied” was erroneously included after the six typical response options for this scale (which range from “Not at all” to “Completely”). Despite this error, responses to this item were strongly correlated with the summed-aggregate of the other three items, *r* = 0.86, and thus were retained for use in this study. Because the number of response options varied across items, responses to each item were standardized independently prior to being mean aggregated to create a composite measure of relationship satisfaction (α = 0.89). Scores on the resulting aggregate ranged from −3.13 (low relationship satisfaction) to 1.01 (high relationship satisfaction).

##### Study 1: sexual satisfaction

Sexual satisfaction was measured with Lawrence and Byers’ (1998; as cited in Byers, 2005) five-item Global Measure of Sexual Satisfaction. Participants rated their sexual relationships on five 7-point bivalent scales: good-bad, pleasant-unpleasant, positive-negative, satisfying-unsatisfying, valuable-worthless. Responses to these items were mean averaged to create a measure of sexual satisfaction (α = 0.97) that ranged from 1 (low sexual satisfaction) to 7 (high sexual satisfaction).

##### Study 1: pornography use

Participants were instructed that pornography use was “intentionally looking at, reading, or listening to: (a) pictures or videos of nude individuals, (b) pictures or videos in which people are having sex, or (c) written or audio material that describes nude individuals, or people having sex.” Participants were told to exclude sexually interactive online and offline behaviors from their reports of pornography use. Following these instructions, participants were asked about their solitary pornography use (“How frequently do you use pornography while alone (i.e., without your partner)?”) and their shared pornography use (“How frequently do you use pornography together with your partner?”). Response options for both items included (1) – “Never”; (2) – “Almost Never”; (3) – “Less than Once a Month”; (4) – “1–3 Times Per Month”; (5) – “1–2 Times Per Week”; (6) – “3–4 Times Per Week”; (7) – “About Once a Day”; (8) – “More than once a day.” Non-use of pornography was common and responses were positively skewed in this sample: 47.00% of the sample reported never using pornography alone (*S* = 1.08, *p* < 0.001) while 54.00% reported never using it with a partner (*S* = 1.57, *p* < 0.001). Reports of shared pornography use by each partner were strongly correlated, *r* = 0.76, *p* < 0.001, and were mean averaged to create a dyadic index of shared pornography use. To aid interpretability, both the measure of solitary pornography use and the measure of shared pornography use were independently standardized for use in the primary analyses described below. In addition, following recommended practice for response surface analysis (RSA; Shanock et al., 2010) the standardized measure of solitary pornography use was re-centered at the midpoint of the scale range.

#### Study 1: Analytic Plan

Associations between pornography use and relationship and sexual satisfaction were examined with a response surface analysis (Shanock et al., 2010) applied to a linear mixed modeling approach to the actor-partner independence model (APIM; Kenny et al., 2006). This was done by testing a series of increasingly complex APIMs (as described below) using the MIXED command in IBM SPSS Statistics 25 (IBM Corporation, 2017). All APIMs were estimated with maximum likelihood estimation so that differences between nested models could be tested with likelihood-ratio tests. The resulting output from the most complex model supported by the data was then subjected to an RSA using the formula’s outlined by Shanock et al. (2010) and plotted with the plotRSA function of the *RSA* package (Schönbrodt and Humberg, 2020) for *R* (R Core Team, 2020). RSA is a better method for testing similarity-dissimilarity effects than traditional approaches using difference scores (Edwards, 1994, 2002; Shanock et al., 2010). Please note that measures of pornography use were standardized before they were re-centered so that the unstandardized fixed effects coefficients for pornography use reported below can be interpreted as relative effect sizes within each respective model. Standard scores were then re-centered at the midpoint of the scale range to facilitate interpretation in the RSA.

The initial model involved the prediction of relationship satisfaction using actors’ and partners’ solitary pornography use and their interaction. In this case, residuals were modeled by nesting partner within couple and estimating an unstructured residual covariance matrix. In the next step, we added shared pornography use as a between-dyad factor to see if the interaction still held while controlling for shared use. Although we did not anticipate interactions with gender based on our theorizing and findings from previous research, we tested gender effects by adding a main effect and interaction components for gender in a following step. Next, we checked to see if quadratic effects for actors’ and partner’s solitary pornography use improved the prediction using log-likelihood ratio tests. The inclusion of these quadratic components is common practice in RSA. In the current study, the addition of these components did not improve model fit (see Supplementary Data Sheet 1) so similarity-dissimilarity effects were examined in the more parsimonious interaction model, which is how such effects have been tested in previous studies involving pornography use (Willoughby et al., 2016; Kohut et al., 2018). These steps were then repeated to examine the associations between pornography use and sexual satisfaction.

**FIGURE 1:**
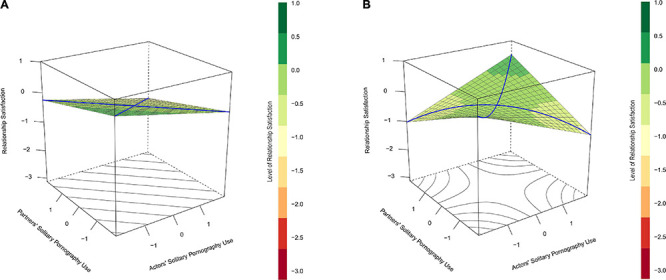
**(A)** Depicts the prediction of relationship satisfaction (vertical axis) with actors’ and partners’ solitary pornography use (horizontal axes) without an interaction between these components. In this case, the plot indicates that as both actors’ and partners’ solitary pornography use increase (the back left-most corner of the plot), participants’ relationship satisfaction decreases. Note that the line of incongruence (the blue line running from left to right of this plot) is a straight line when no interaction is present in the data. The lack of curve in this line indicates the absence of similarity-dissimilarity effect in the data. In contrast, **(B)** depicts the hypothesized similarity-dissimilarity effect by illustrating the prediction of relationship satisfaction with an interaction between actors’ and partners’ solitary pornography use. Unlike the illustration in **(A)**, this plot indicates that participants’ relationship satisfaction decreases as their own solitary pornography use (i.e., actors’ solitary pornography use) increases, but only when their partners’ solitary pornography use is low. Furthermore, the line of incongruence in this example is curved rather than straight, which indicates that a similarity-dissimilarity effect is present in the data. With respect to this line, relationship quality is lower at both extreme ends, when one partner uses pornography alone very frequently and the other does not, than at the midpoint, when both partners use pornography alone at a modest frequency.

To understand RSA it is useful to recognize that all general linear models in which two predictors are regressed on an outcome can be thought of as a prediction surface which describes the anticipated outcome (*Z*) at different levels of each predictor (*X* and *Y*). In the absence of an interaction between predictors (*Z*′ = *b*_0_ + *b*_1_*X* + *b*_2_*Y*) the prediction surface is flat plane where the “tilt” or “orientation” is determined by the main effects of the predictors (defined by *b*_1_ and *b*_2_; see Figure 1A). In the presence of an interaction though (*Z*′ = *b*_0_ + *b*_1_*X* + *b*_2_*Y* + *b*_3_*XY*), the specific associations between each predictor and the outcome vary as a function of the other predictor, and this results in a curvilinear distortion of the prediction surface, defined by *b_3_*, that can take one of several constrained forms (for one such example see Figure 1B).

An RSA builds on this logic by using simple algebra to recombine coefficients (and their associated standard errors) from an expanded polynomial regression of the form, *Z*′ = *b*_0_ + *b*_1_*X* + *b*_2_*Y* + *b*_3_*X*^2^ + *b*_4_*XY* + *b*_5_*Y*^2^). This recombination is done to test four new parameters that describe the attributes of two arbitrary lines that run through the prediction surface: the line of congruence (defined as the line where *X* = *Y*) and the line of incongruence (defined as the line where *X* = –*Y*). With respect to the current study, the line of congruence describes the predicted levels of relationship (and sexual) satisfaction for cases where partners report the same frequencies of solitary pornography use or non-use (actors’ solitary pornography use = partners’ solitary pornography use). In contrast, the line of incongruence defines the predicted levels of relationship (and sexual) satisfaction when partners’ patterns of pornography use range from cases where participants use pornography alone more than once a day (actors’ solitary pornography use = 1.97) but their partners do not use pornography alone (partners’ solitary pornography use = −1.97), through cases where both participants and their partners use pornography about once a week (actors’ solitary pornography use = 0 and partners’ solitary pornography use = 0), to cases where the participants do not use pornography alone (actors’ solitary pornography use = −1.97) and but their partners do so more than once a day (partners’ solitary pornography use = 1.97). The line of incongruence plots a course on the prediction surface that ranges from one form of extreme dissimilarity, to a point of similarity, to another form of extreme dissimilarity (see the blue line running from the left to right side of Figure 1B).

The goal of RSA is to test slopes (indicated by coefficients a_1_ and a_3_) and curvilinear components (indicated by coefficients a_2_ and a_4_) for the line of congruence and the line of incongruence, and each component has a specific interpretation. With respect to the focal predictions of this study, coefficient *a*_4_ is most relevant because it describes the curvature along the line of incongruence. When *a*_4_ is significant it means that a similarity-dissimilarity effect is present in the data. If this coefficient is negative, it describes a convex shape. In the context of the current study a convex curve along the line of incongruence would mean that predicted satisfaction scores are higher when partners both report using pornography alone about once a week than when they report extreme dissimilarity in solitary pornography use (see Figures 1A,B). A significant slope along the line of incongruence denoted by coefficient a_3_, describes the degree of tilt that is present in the curve. Depending on the direction of this slope, this will enhance the magnitude of the effect of one type of extreme dissimilarity while diminishing the magnitude of the other (e.g., relationship satisfaction is lower when participants frequently use pornography alone and their partners do not than when participants do not use pornography alone but their partners do so frequently).

### Study 1: Results

Correlations between primary measures can be found in Table 1. Notable correlations existed between men’s and women’s reports of relationship satisfaction, *r* = 0.65, *p* < 0.001, sexual satisfaction, *r* = 0.52, *p* < 0.001, and shared pornography use, *r* = 0.76, *p* < 0.001, as well as between the within-subject reports of sexual and relationship satisfaction provided by men, *r* = 0.66, *p* < 0.001, and women, *r* = 0.70, *p* < 0.001.

**FIGURE 2:**
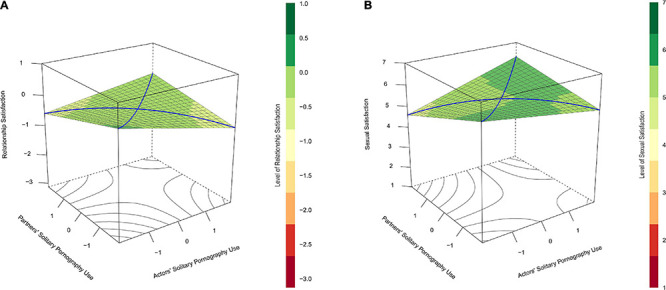
**(A)** Depicts predicted relationship satisfaction (vertical axis) as a function of actors’ (*x*-axis) and partners’ (*y*-axis) frequencies of solitary pornography use and their interaction for cases that reported mean levels of shared pornography use in Study 1. **(B)** Does the same for the prediction of sexual satisfaction. In both cases, satisfaction scores tended to be lowest when couple members were most dissimilar in their solitary pornography use (left- and right-most corners of the plots), though this effect did not remain statistically significant when predicting relationship satisfaction **(A)** once gender was controlled for. With respect to sexual satisfaction **(B)**, a similarity-dissimilarity effect is evident: the curvilinear component of the line of incongruence was significant and region of significance tests indicated a participant’s own pornography use was negatively related to their sexual satisfaction, but only among participants who’s partners almost never used pornography (partners’ solitary pornography use <–1.74).

The initial model predicted relationship satisfaction using actors’ and partners’ reports of solitary pornography use and their interaction. In subsequent steps, model fit was improved, *χ*^2^ (1) = 8.46, *p* = 0.004, by adding shared pornography use as a between-dyad covariate, and gender as a within-dyad factor, *χ*^2^ (1) = 15.96, *p* < 0.001. Consistent with H1a, frequency of shared pornography use was significantly related to relationship satisfaction, *b* = 0.17, *p* = 0.004, such that couples who reported more frequent shared pornography use reported higher relationship satisfaction. However, with respect to the expected similarity-dissimilarity effect (H2a), the positive interaction term was in the anticipated direction but dropped from significant, *b* = 0.10, *p* = 0.042, to non-significant, *b* = 0.10, *p* = 0.061, when the main effect for gender was added to the model (see Figure 2A and Table 2). Adding further interactions between gender and the other components of the model did not significantly improve model fit, *χ*^2^ (4) = 3.31, *p* = 0.508.

**TABLE 2:** Linear mixed models predicting relationship and sexual satisfaction for Study 1 (*N* = 200 couples).

	**Relationship Satisfaction**		**Sexual Satisfaction**	
	***b***	***p***	***b***	***p***
**Fixed Effects**				
Intercept	−0.24	0.029	5.63	>0.001
Actors’ Solitary Porn. Use	−0.05	0.493	0.11	0.305
Partners’ Solitary Porn. Use	−0.04	0.608	−0.09	0.399
Actors’ by Partners’ Solitary Porn. Use	0.10	0.061	0.16	0.043
Shared Porn. Use	0.17	0.004	0.23	0.014
Gender	−0.12	>0.001	−	−

Sexual satisfaction was analyzed in the same way. In this case, adding the main effect for shared pornography use significantly improved fit, *χ*^2^ (1) = 6.08, *p* = 0.014, but adding the main effect for gender did not *χ*^2^ (1) = 3.10, *p* = 0.078. Within this model, the interaction between actor’s and partners’ solitary pornography use was significant when predicting sexual satisfaction, *b* = 0.16, *p* = 0.043, as was the frequency of shared pornography use, *b* = 0.23, *p* = 0.014 (see Table 2 and Figure 2B). The RSA (Shanock et al., 2010) of the solitary pornography use components of this model further revealed significant curves along the lines of congruence, *a*_2_ = 0.16, *p* = 0.043, and incongruence, *a*_4_ = −0.16, *p* = 0.043, as well as a positive slope along the line of incongruence, *a*_3_ = 0.20, *p* = 0.009. In sum, as we predicted, sexual satisfaction tended to be higher among participants who reported more shared pornography use (H1b) and lower among participants who reported high dissimilarity in their frequencies of solitary pornography use (H2b). However, the significant slope for *a*_3_ indicated that the effects of dissimilarity on sexual satisfaction were particularly pronounced in cases where participants used little to no pornography alone but their partners frequently used pornography alone (see Figure 2B). Model fit was not improved further by adding interactions between gender and the other components of the model, *χ*^2^ = 0.964, *p* = 0.915.

Additional region of significance tests (Preacher et al., 2018) for simple slopes of actors’ solitary pornography use at different values of a partners’ solitary pornography use indicated a break point at −1.74. In this case, participants’ own pornography use was negatively related to their sexual satisfaction, but only among participants whose partner almost never used pornography, otherwise, pornography use was unrelated to their sexual satisfaction.

### Study 1: Discussion

The results of Study 1 extended the previously established positive associations between shared pornography use and relationship quality (e.g., open sexual communication and closeness; see Kohut et al., 2018) to measures of relationship and sexual satisfaction. The results suggest that relationship and sexual satisfaction was higher among couples who use pornography together on a more frequent basis. The associations between similarity-dissimilarity in solitary pornography use and satisfaction, however, were more nuanced. Sexual satisfaction varied, as predicted, by the degree of similarity-dissimilarity in partners’ frequencies of solitary pornography use. Specifically, sexual satisfaction was lower when one partner used pornography alone while the other did not, and this effect was more pronounced among the partner who did not use pornography alone than among the partner who used pornography alone. With respect to relationship satisfaction, the pattern of effects was similar but weaker, and did not remain after an effect for gender was added to the model. This gender difference in relationship satisfaction was unexpected as previous analyses of this dataset have not found gender effects in the other measures of relationship quality once indicators of pornography use were controlled for (Kohut et al., 2018).

## Study 2: Attitudes Toward One’s Own Pornography Use

Although the correlates of shared pornography use are important in their own right, the theoretical mechanism underlying the correlates of similarity-dissimilarity in solitary pornography use is of particular interest to us. One possibility is that such associations stem from partner dissimilarity in attitudes toward pornography use. Within the literature, attitudes toward pornography use have been operationalized in a variety of different ways including the degree that people believe that pornography is arousing (Haavio-Mannila, 2003), the degree that they believe pornography is exciting and entertaining (Træen et al., 2004), the degree that they believe that it is beneficial or harmful (Træen et al., 2004; Poulsen et al., 2013) and the degree that they approve or disapprove of the use of others (Carroll et al., 2008). Men have notably more positive attitudes toward pornography use than women in that they tend to report that using pornography is more exciting, entertaining, and self-enhancing (Træen et al., 2004), and are also more approving of the use of others (Carroll et al., 2008, 2017). People with more positive attitudes toward pornography also tend to report using it more frequently (Træen et al., 2004; Poulsen et al., 2013), and because men are more likely to use pornography than women (Petersen and Hyde, 2010), it is likely that partners in many heterosexual relationships have dissimilar attitudes in this regard. Given previous findings concerning sexual similarity-dissimilarity (Eysenck and Wakefield, 1981; Smith et al., 1993; Purnine and Carey, 1999; Montoya and Horton, 2013), it seemed likely that partner similarity-dissimilarity in attitudes toward pornography use should also be related to relationship and sexual satisfaction. Finding such effects would lend credit to the possibility that similarity-dissimilarity in constructs related to pornography use may be statistically confounded with the similarity-dissimilarity effects of solitary pornography use (RQ1).

To the extent that attitudes toward using pornography oneself serve as a marker for actual pornography use (more positive attitudes reflect more pornography use), a harm-focused exposure-based approach would argue that positive attitudes should correlate with a decline in relationship and sexual satisfaction over time. To this point, some existing longitudinal studies have found that pornography use precedes changes in relationship functioning (Perry, 2017a, b; Wright et al., 2017), though few of these studies have been dyadic in nature and at least some evidence indicates that relationship dysfunction can precede changes in pornography use (Muusses et al., 2015). To our knowledge no longitudinal studies have examined the role similarity-dissimilarity in partners’ pornography use – or more relevantly here, partners’ attitudes toward using pornography themselves – as an important moderating influence.

From an attitudinal similarity perspective, however, alternative expectations for the association between attitudes toward pornography use and changes in relationship and sexual satisfaction are possible. In a new couple, for example, one might expect that full awareness of differences in attitudes between partners may have yet to emerge, especially with respect to attitudes toward sexual interests like pornography use. If that were true, the magnitude of expected positive interaction between partners’ attitudes toward pornography use could increase over time, reflecting increasingly negative impacts of dissimilarity in attitudes (relative to similarity in attitudes) as partners learn more about one another. On the other hand, in the West, sexual interactions frequently begin in the early stages of relationships, with few people waiting for the commitment of marriage (Wu et al., 2017), so partners may become aware of similarities and differences in each other’s attitudes toward pornography use (or other closely related attitudes) relatively early on. In such circumstances, the magnitude of the associations between the relationship and sexual satisfaction and the interaction between partners’ attitudes toward using pornography themselves could either remain constant across time or decrease over time, depending largely on the (a) stability of attitudes, and (b) the stability of the relationship between attitudes toward pornography and sexual satisfaction.

Such questions were examined with a subset of data drawn from an 8-wave longitudinal study of American newlyweds that tracked relationship and sexual satisfaction over three and a half years in approximately 6-month intervals. Data were restricted to assessments at waves 5 through 8 because this study did not measure attitudes toward pornography use until wave 5, roughly 2 years from study initiation. Unfortunately, this measure of attitudes was not repeated in subsequent waves, which restricted the nature of temporal questions that could be examined with this data. With the previous findings in mind, we generally expected that partners’ attitudes would moderate the associations between actors’ attitudes toward using pornography themselves and relationship and sexual satisfaction such that satisfaction would be lower when actors and partners were discrepant in their attitudes toward using pornography. Because existing similarity-dissimilarity effects of pornography use have generally not been moderated by gender, we did not believe that the actor, partner, or the actor by partner interaction effects of attitudes would be further moderated by gender in this case either. Given the lack of the previous time-relevant dyadic research to draw on, coupled with divergent theoretical expectations, we had no firm expectations about whether the anticipated interaction effects would be further moderated by time (registered materials: https://osf.io/846vp; data and syntax^3, 4^). Details of related ancillary research questions concerning curvilinear associations between attitudes toward pornography use and relationship quality can be found in Supplementary Data Sheet 1.

### Study 2: Method

#### Study 2: Participants

Participants were drawn from a larger eight-wave longitudinal study of 135 American newlywed couples. Data analysis was restricted to data from waves 5 through 8 of the study because the assessment of attitudes toward using pornography did not occur until the 5th wave. In the latter half of this study, only 154 members of 77 couples had provided data that could be analyzed (recruitment details can be found in McNulty, 2016).

Of the *N* = 154 participants who had complete assessments of attitudes toward their own pornography use and at least one of the two dependent variables for both themselves and their partners, one hundred and forty-six (94.81%) reported relationship satisfaction at least twice; 120 (77.92%) reported relationship satisfaction at least three times, and eighty reported relationship satisfaction (51.95%) on all measurement occasions (reports of sexual satisfaction followed a similar pattern). Missing data analysis indicated that attitudes toward pornography use were somewhat more positive among those with missing relationship and sexual satisfaction data at wave 2, but there was no evidence of non-random missingness. As a consequence, we assumed that data were “Missing at Random” rather than “Missing at Complete Random” and proceeded with the planned linear mixed models (described below).

At study baseline, approximately 2 years before the 5th wave of the study, the husbands examined here were 25.79 years old (*SD* = 4.13) and had completed 16.19 years of education (*SD* = 2.08). The median of husbands’ reported income range was $20,001 to $25,000 per year. Wives were 23.95 years old (*SD* = 3.28) and had completed 17.25 years of education (*SD* = 1.60). The median of wives’ reported income ranged from $10,001 to $15,000 per year. Eighty-seven percent of husbands and 91% of wives identified as Caucasian. Husbands who were retained for analysis were significantly more educated than those who were not, *t*(133) = 2.51, *p* = 0.013, but these groups did not differ in age, *t*(133) = 0.33, *p* = 0.745, race, *χ*^2^(8) = 13.06, *p* = 794, or income, *t*(132) = −1.30, *p* = 0.197. Wives that were retained for analysis did not differ from those who were excluded in terms of education, *t*(133) = −0.46, *p* = 0.648, age, *t*(133) = 0.95, *p* = 0.346, race, *χ*^2^(3) = 2.74, *p* = 434, and income, *t*(132) = −0.19, *p* = 0.847.

#### Study 2: Materials and Procedure

As part of the broader aims of the original study from which these data were drawn, couples attended a laboratory session. Before that session, they were mailed a packet of questionnaires to complete at home and bring to their appointment. This packet included a consent form approved by the local human subjects review board, questionnaires beyond the scope of the current analyses (e.g., personality, stress, self-esteem, etc.), and a letter instructing participants to complete all questionnaires independently of their partners and to bring their completed questionnaires to their upcoming laboratory session at which they completed other tasks that are not relevant to the current analyses (see McNulty and Russell, 2010; McNulty, 2016). Couples were paid US$80 for participating in this phase of the study.

At approximately 6- to 8-month intervals subsequent to the initial assessment, couples were re-contacted by phone or email and again mailed further questionnaire packets. At the fifth wave of assessment, the packet contained questions about relationship satisfaction, sexual satisfaction, and attitudes toward pornography use. The sixth, seventh, and eighth wave of assessment contained the same measures of relationship and sexual satisfaction but not attitudes toward pornography use. Couples were mailed a US$50 check for participating in each of these follow-up phases. Means and standard deviations for the following measures can be found in Table 3.

**TABLE 3:** Summary of the correlations, means, and standard deviations of the focal variables for Study 2 (*N* = 77 couples).

	**1**	**2**	**3**	**4**	**5**	**M**	**SD**
1. Relationship Satisfaction Male						40.08	4.95
2. Relationship Satisfaction Female	0.49**					39.62	6.74
3. Sexual Satisfaction Male	0.47**	0.43**				140.22	27.06
4. Sexual Satisfaction Female	0.40**	0.64**	0.54**			139.39	23.94
5. Attitudes Toward Porn Male	−0.24*	−0.30**	−0.26*	−0.26*		3.47^1^	2.01
6. Attitudes Toward Porn Female	–0.07	–0.08	–0.09	–0.14	0.44**	2.27	1.68

*Signifies significant correlations *p* < 0.05; **signifies significant correlations, *p* < 0.01.

*^1^Women report significantly more negative attitudes toward pornography than men, *p* < 0.001.*

*For interpretability, all *M* and *SD* are presented in the original scale metric.*

##### Study 2: relationship satisfaction

Relationship satisfaction was assessed with the Quality Marriage Index (Norton, 1983) at waves 5 through 8. It consists of five items that ask participants the extent to which they agree or disagree with general statements about their marriage (e.g., “We have a good relationship”) on a scale from “Very strong disagreement” (1) to “Very strong agreement” (7), and one item that asks spouses to answer the question “All things considered, how happy are you with your marriage?” on a scale from “Very unhappy” (1) to “Perfectly happy” (10). Scores were summed so that higher scores indicated more satisfaction. Reliability was high; Cronbach’s α was above 0.90 for both husbands and wives across all waves.

##### Study 2: sexual satisfaction

The degree of spouses’ sexual satisfaction was assessed at waves 5 through 8 with the Index of Sexual Satisfaction (Hudson, 1998). This inventory measures partners’ satisfaction with their sexual relationship by asking them to indicate the extent to which 25 statements described their current sexual relations with their partner (e.g., “I think that our sex is wonderful”; “Our sex is monotonous”) on a scale from “None of the time” (1) to “All of the time” (7). Responses to these items were reversed where appropriate and summed to form aggregate scores which ranged from 25 to 175, with higher scores indicating higher levels of sexual satisfaction. Internal consistency of this measure was high (α > 0.90) across all study waves for both partners.

##### Study 2: attitudes toward one’s own pornography use

Participants’ attitudes toward using pornography themselves were assessed with a single item on a broader scale of sexual attitudes (Wenner et al., 2011) at wave 5 of the study. This item asked each member of the couple to report the extent to which they agreed with the following statement: “I enjoy viewing pornography,” using a scale ranging from “Very strong disagreement” (1) to “Very strong agreement” (7). As before, attitudes toward pornography use were standardized and then re-centered at the midpoint of the range for use in the analyses described below.

#### Study 2: Analytic Plan

Both relationship and sexual satisfaction were analyzed with RSA applied to a linear mixed-modeling approach to the actor-partner growth model where actors’ and partners’ attitudes toward pornography use, their interaction, and gender served as time invariant predictors of either relationship or sexual satisfaction measured over 4 waves (waves 5 through 8). The initial models considered only the actor and partner effects for attitudes toward pornography use, as well their interaction, and in the following steps, fixed effects of gender and time were considered in subsequent models. Following Kenny et al.’s (2006) advice for repeated measures dyadic data, we modeled correlated errors in the residuals by nesting time crossed with partner within couples and constraining the resulting covariance matrix with a heterogeneous auto-regressive structure. This approach allows residuals to correlate between partners and across time and assumes larger correlations between measures that are more temporally proximate. Analyses were conducted with the MIXED command in IBM SPSS Statistics 25 (IBM Corporation, 2017) and all models were estimated with maximum likelihood estimation so that differences between nested models could be tested.

### Study 2: Results

Baseline correlations between primary measures can be found in Table 3. Moderate correlations existed between relationship partners’ reports of relationship satisfaction, *r* = 0.49, *p* < 0.001, and sexual satisfaction, *r* = 0.54, *p* < 0.001. Importantly, small negative correlations also emerged between men’s attitudes toward using pornography themselves and both their own, *r* = −0.25, *p* = 0.032, and their partners’ relationship satisfaction, *r* = −0.30, *p* = 0.007. The same was true with respect their own, *r* = −0.26, *p* = 0.023, and their partners’ sexual satisfaction, *r* = −0.26, *p* = 0.020.

**FIGURE 3:**
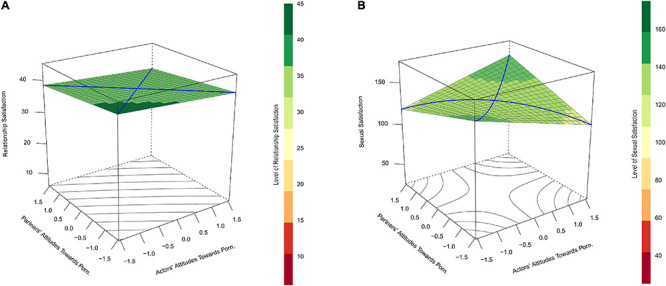
**(A)** Depicts predicted relationship satisfaction (vertical axis) as a function of actors’ (*x*-axis) and partners’ (*y*-axis) attitudes toward using pornography themselves and the interaction between these variables in Study 2. **(B)** Does the same for the prediction of sexual satisfaction. In **(A)**, the lack of significant interaction results in a flat prediction surface where partners with dissimilar attitudes toward pornography are not notably lower in relationship satisfaction than partners who are more similar in their attitudes. In **(B)**, sexual satisfaction scores tended to be lowest in cases in which couple members were most dissimilar in their attitudes toward pornography (left- and right-most corners of the plots). According to regions of significance tests, participants’ attitudes toward using pornography themselves were negatively related to their own sexual satisfaction if their partners indicated any degree of dislike of pornography (partners’ attitudes toward pornography use <–0.36) but positively related to their sexual satisfaction when their partners indicated agreement or strong agreement with personally enjoying pornography (partners’ attitudes toward pornography use >0.68).

The initial model predicted relationship satisfaction with actors’ and partners’ reports of attitudes toward pornography use and their interaction. Contrary to our expectations, the interaction effect was not significant, *b* = 0.03, *p* = 0.933 (see Table 4). The lack of interaction resulted in a flat prediction surface which does not indicate similarity-dissimilarity effects (see Figure 3A). Neither of the subsequent models involving gender, *χ*^2^ (4) = 5.46, *p* = 0.243, and time, *χ*^2^ (4) = 4.39, *p* = 0.356, improved model fit further.

**TABLE 4:** Linear mixed models predicting relationship and sexual satisfaction for Study 2 (*N* = 77 couples).

	**Relationship Satisfaction**		**Sexual Satisfaction**	
	**b**	**p**	**b**	**p**
**Fixed Effects**				
Intercept	39.17	>0.001	136.2	>0.001
Actors’ Attitudes Toward Porn (ATP)	−0.63	0.133	−0.02	0.991
Partners’ ATP	−1.01	0.011	1.19	0.521
Actors’ ATP by Partners’ ATP	0.03	0.933	8.11	>0.001

Sexual satisfaction was predicted by actors’ and partners’ reports of attitudes toward pornography use and their interaction. As we suspected, this model indicated a significant positive interaction between actors’ and partners’ attitudes toward pornography use, *b* = 8.11, *p* < 0.001 (see Figure 3B and Table 4). A RSA further indicated significant curves along the lines of congruence, *a*_2_ = 8.11, *p* < 0.001, and incongruence, *a*_4_ = −8.11, *p* < 0.001, but no significant slopes along these lines: *a*_1_ = 1.17, *p* = 0.711, and a_3_ = −1.21, *p* = 0.507. Such findings suggested a similarity-dissimilarity effect for partners’ attitudes toward pornography use when predicting sexual satisfaction. The lack of slope for a_3_ in this analysis indicated that the effect of similarity-dissimilarity was the same regardless of which partner’s attitudes toward pornography were positive and which were negative.

Region of significance tests indicated significant slopes for actors’ attitudes toward pornography use when partners’ attitudes toward pornography use were less than −0.36 and greater than 0.68. These results suggested that participants’ attitudes toward using pornography themselves were negatively related to their sexual satisfaction if their partners indicated any degree of dislike of pornography but positively related to their sexual satisfaction when their partners indicated agreement or strong agreement with personally enjoying pornography.

Consistent with our prediction, adding a gender component and interactions between gender and the other fixed effects in the model did not improve model fit further, *χ*^2^ (4) = 3.23, *p* = 0.520. Interestingly, adding a time component and interactions between time and the other fixed effects in the model also failed to improve model fit, *χ*^2^ (4) = 3.23, *p* = 0.495, indicating that the association between similarity-dissimilarity in attitudes toward pornography and sexual satisfaction did not change from wave 5 to wave 8, which occurred approximately 18 months later.

### Study 2: Discussion

The results of Study 2 partially confirmed our primary expectations; like Study 1 a significant positive interaction between actors’ and partners’ attitudes toward using pornography themselves was found when predicting sexual satisfaction but not relationship satisfaction. Unfortunately, because pornography use was not measured in this study, it was not possible to test this explanatory mechanism for the interactive effects of partners’ solitary pornography use on sexual satisfaction directly.

This study also found that similarity-dissimilarity in attitudes toward pornography use measured some years after marriage remained predictive of sexual satisfaction 18 months after the attitudes were measured. Moreover, the statistical effect of similarity-dissimilarity appeared to be relatively constant across time, neither increasing in magnitude nor decreasing. We are inclined to believe that such findings reflect a degree of stability within partner similarity-dissimilarity in attitudes toward pornography use, which may be entrenched in a broader network of similar-dissimilar values. We also note that this is not a pattern of results that we would expect from a harm-focused effect-based approach. However, without further assessments of attitudes toward pornography use at subsequent waves of data collection, broader measures of sexual value dissimilarity, or measures of actual pornography use, the best interpretation of this pattern of association remains uncertain.

## Study 3: Erotophobia-Erotophilia and Sexual Preferences

Although suggestive, the results of Studies 1 and 2 are far from definitive. Of particular importance, the findings from Study 1 should not be taken as strong evidence of a conceptual extension of the correlates of similarity-dissimilarity in solitary pornography use or shared pornography use. The data used in Study 1 came from the same sample employed by Kohut et al. (2018), who reported nearly identical patterns of results with respect to sexual communication and interpersonal closeness. To further substantiate these findings, we sought to replicate them by testing H1 and H2 in an independent cross-sectional sample of *N* = 207 romantic dyads that was collected for a project involving sexual ideal preferences (Balzarini et al., 2021). In this sample, we expected to find clear evidence that shared pornography use would be associated with both relationship and sexual satisfaction (H1) and that dissimilarity in solitary pornography use would be related to lower relationship and sexual satisfaction (H2).

An additional goal of this study was to strengthen the elucidation of theoretical mechanisms underlying the similarity-dissimilarity effect of solitary pornography use by further examining RQ1. Unlike Study 2, the data used for Study 3 included both measures of pornography use and relevant individual difference dimensions which allowed us to test whether or not similarity-dissimilarity in pornography use added to the prediction of sexual/relationship satisfaction once other patterns of similarity-dissimilarity were statistically controlled. If similarity-dissimilarity in solitary pornography use did not emerge under these circumstances, it would suggest either that similarity-dissimilarity in other characteristics mediate the causal impact of pornography on relationship quality, or potentially, that the associations between pornography use and relationship quality may be spurious.

Individual differences in couple members’ erotophobia-erotophilia are of considerable conceptual relevance to understanding the connections between pornography use and relationship quality because erotophobia-erotophilia is reliably correlated with pornography use (Fisher et al., 1988). Moreover, previous research has shown that similarity-dissimilarity in erotophobia-erotophilia is related to sexual satisfaction (Smith et al., 1993). If similarity-dissimilarity in partners’ erotophobia-erotophilia is implicated in the effects of similarity-dissimilarity in solitary pornography use, we would expect to find a positive interaction between actors’ and partners’ erotophobia-erotophilia when predicting relationship and sexual satisfaction. Further, if erotophobia-erotophilia is a major contributor to relationship functioning – either because it represents an ultimate cause and pornography use is a mediating process or because it is a proximal cause that either mediates or creates spurious associations between pornography use and relationship functioning – actors’ and partners’ solitary pornography use and the interaction between these variables should not add to the prediction of sexual/relationship satisfaction once actors’ and partners’ erotophobia-erotophilia, and their interaction, are statistically controlled.

As an alternative to erotophobia-erotophilia, it is also known that similarity-dissimilarity in partners’ sexual preferences (e.g., “My preferred time for having sex is in the morning”) are related to sexual satisfaction (Purnine and Carey, 1999). From this perspective, the data used for the current analyses are particularly relevant as they come from a dyadic study that was designed to determine if discrepancies in partners’ sexual ideals are associated with various aspects of relationship quality. To this end, members of participating couples were each asked to indicate their own sexual ideal preferences across a diverse set of 30 items, none of which specifically involved pornography use. Because differences in pornography use may reflect wider differences in personally held sexual ideal preferences within relationships, controlling for differences in personally held sexual ideals may also reduce or eliminate the associations between pornography use and relationship and sexual satisfaction.

In sum, Study 3 had two research goals. First, we wished to determine if the associations between similarity-dissimilarity in solitary pornography use, shared pornography use, and relationship and sexual satisfaction would replicate in an independent dyadic sample. Second, we wished to determine if similarity in erotophobia-erotophilia and/or sexual ideal preferences could statistically “explain” these associations (registered materials: https://osf.io/h8agx; data and syntax^5^). Details of related ancillary research questions concerning curvilinear associations between pornography use and relationship quality can be found in Supplementary Data Sheet 1.

### Study 3: Method

#### Study 3: Participants

This sample was recruited by *Qualtrics Panel LLC*. To this end, *Qualtrics* contacted panel members with the opportunity to participate in a study whose stated purpose was to better understand sexual ideals and to assess the associations between sexual ideal discrepancies and relationship functioning. To be eligible, individuals were required to be at least 18 years of age, be fluent in English, to have an active *Qualtrics Panel* account, to be involved in a romantic relationship of at least 4 months, and to have a romantic partner willingly complete the survey. These inclusion criteria were confirmed through a screening process conducted by *Qualtrics Panel LLC*, and subsequently reconfirmed by participants’ responses to the demographic questionnaire.

A total of 2,050 individuals accessed the online study, and of those, 1,843 were removed because one or both partners: did not consent to participate (12.93%, *n* = 265), failed to meet the inclusion criteria (22.73%, *n* = 466), failed an attention check (25.37%, *n* = 520), did not complete the study in full (28.20%, *n* = 578), or because our quota was reached (0.20%, *n* = 4). The final sample was composed of *N* = 207 heterosexual couples (*N* = 414 individuals). Retained couple members were primarily middle-aged (*M* = 45.81), Caucasian (84.54%), monogamous (88.89%), and married (88.41%). Compared to the participants in intact couples with complete data who were not included in this study, the retained sample reported significantly higher solitary pornography use, *t*(237) = 2.85, *p* = 0.005. These two subsamples did not differ with respect to age, *t*(237) = 0.87, *p* = 0.387, race, *F*(1,476) = 2.99, *p* = 0.084, relationship orientation, *F*(1,476) = 0.52, *p* = 0.473, relationship status, *F*(1,476) = 0.19, *p* = 0.667, relationship satisfaction, *t*(236) = −1.08, *p* = 0.281, sexual satisfaction, *t*(236) = −1.89, *p* = 0.070, or shared pornography use, *t*(237) = −0.09, *p* = 0.928.

#### Study 3: Materials and Procedure

Eligible parties followed a link to a webpage which presented the Letter of Information and informed consent. Participants were first asked to fill out a questionnaire assessing demographic information. Then, participants were asked to build a mental picture of their ideal sexual partner and to indicate how important each of 30 traits was to their concept of an ideal sexual partner. After this exercise, participants rated the extent to which they believed their actual partner met these 30 ideals. Participants were then asked about their perceptions of their actual partners’ ideals and the extent to which they believed they met their partners’ ideals. Next, participants responded to a series of measures meant to examine relationship functioning correlates (e.g., relationship and sexual satisfaction, perceived likelihood of relationship dissolution) of similarity-dissimilarity in sexual ideal preferences, potential moderators of these associations (e.g., erotophobia-erotophilia, implicit theories of relationships, motivations for sex), and questions about their solitary pornography use and their shared pornography use with their partner. Once all questionnaires were complete, participants were forwarded to a page where they were provided with debriefing information and token compensation for taking part in the study. Means and standard deviations for the following measures can be found in Table 5.

**TABLE 5:** Summary of the correlations, means, and standard deviations of the focal variables for Study 3 (*N* = 207 couples).

	**1**	**2**	**3**	**4**	**5**	**6**	**7**	**8**	***M***	***SD***
1. Relationship Satisfaction Male									7.61^1^	1.84
2. Relationship Satisfaction Female	0.76**								7.28	2.06
3. Sexual Satisfaction Male	0.77**	0.69**							6.06^1^	1.32
4. Sexual Satisfaction Female	0.63**	0.84**	0.76**						5.81	1.45
5. Solitary Porn. Use Male	−0.16*	−0.21**	−0.18*	−0.20**					2.96^1^	1.77
6. Solitary Porn. Use Female	–0.05	–0.03	–0.06	–0.01	0.38**				2.01	1.33
7. Shared Porn. Use Male	0.08	0.08	0.20**	0.14*	0.42**	0.39**			1.99	1.11
8. Shared Porn. Use Female	–0.03	0.05	0.13	0.15*	0.37**	0.44**	0.86**		1.95	1.22
9. Mean Shared Porn. Use	0.02	0.07	0.17*	0.15*	0.41*	0.43*	0.96**	0.97**	1.97	1.13

*Signifies significant correlations *p* < 0.05; **signifies significant correlations, *p* < 0.01.

*^1^Men report significantly higher relationship satisfaction, *p* < 0.001, sexual satisfaction, *p* < 0.001, and pornography use, *p* < 0.001, than women.*

*For interpretability, all *M* and *SD* are presented in the original scale metric.*

##### Study 3: relationship satisfaction

Three items from the Investment Model Scale (Rusbult et al., 1998) were used to assess relationship satisfaction (e.g., “I feel satisfied with our relationship”). Participants responded with 9-point scales that ranged from “do not agree at all” (1) to “agree completely” (9). Responses to these items were mean aggregated (α = 0.95), with higher scores indicating more relationship satisfaction.

##### Study 3: sexual satisfaction

As with Study 1, sexual satisfaction was measured with Lawrence and Byers’ instrument (1998; as cited in Byers, 2005). Responses were mean aggregated (α = 0.97) with higher scores indicating more sexual satisfaction (range: 1–7).

##### Study 3: pornography use

Pornography use was assessed with the same 2-items used in Study 1 to obtain information about the frequency of participants’ solitary pornography use and their shared pornography use with their partner. However, in this case, definitions of pornography use were not provided to participants. Response options ranged from “Never” (1) to “More than once a day” (8). As with Study 1, non-use of pornography was common and responses were positively skewed in this sample: 39.61% of the sample reported never using pornography alone (*S* = 0.94, *p* < 0.001) while 46.62% reported never using it with a partner (*S* = 1.25, *p* < 0.001). As before, partners’ respective reports of shared pornography use were mean averaged and both the measure of solitary pornography use and shared pornography use were standardized and re-centered at the midpoint of the scale range for use in the RSA analyses described below.

##### Study 3: erotophobia-erotophilia

The short-form of the Sexual Opinion Survey (Fisher et al., 1988) was used to assess erotophobia-erotophilia. Participants were asked to indicate the extent they agreed or disagreed with five statements such as, “Masturbation can be an exciting experience” and “It would be emotionally upsetting to me to see someone exposing themselves publicly” (α = 0.67). Participants responded using a 7-point Likert-like scale ranging from “strongly agree” (1) to “strongly disagree” (7). Responses were mean aggregated with reverse coding were applicable so that higher scores indicated more erotophilia, and then standardized.

##### Study 3: sexual ideal preferences

Participants were asked to mentally construct an ideal sexual relationship and then indicate how important each of 30 items was for understanding that relationship. Items included specific partner traits (e.g., “Ideal sexual partner is kinky”), optimal aspects of sexual encounters (e.g., “Ideal sexual encounter would involve dirty talk”), and other characteristics of one’s ideal relationship with a sexual partner (e.g., “Go on dates with ideal sexual partner”). Participants responded to the items with 7-point scales that ranged from “very unimportant” (−3) to “very important” (3). Responses were subjected to an exploratory factor analysis using maximum-likelihood and an oblimin rotation. A parallel analysis indicated that no more than six factors should be extracted though a five factor solution was most interpretable. The five factor solution explained 40% of the variance and had reasonable fit, TLI = 0.91, RMSEA = 0.04; 90% CI [0.04, 0.05]. The resulting factors indicated discrete preferences for aggressive sex (e.g., spanking, hair-pulling, etc.), a loving partner and relationship (e.g., loving, supportive, etc.), pornographic sex (e.g., swallowing ejaculate, anal sex, etc.), specific partner demographic characteristics (e.g., ethnicity, religiosity, etc.), and holistic somatic stimulation (e.g., nipple stimulation, tickling, etc.). Factor scores were calculated for each factor using the regression method and then standardized. Weighted composite reliabilities for regression factors scores (Beauducel et al., 2016) ranged from adequate to good: aggressive sex, *R*_*R*_ = 0.90; a loving partner and relationship, *R*_*R*_ = 0.88; pornographic sex, *R*_*R*_ = 0.79; specific partner characteristics, *R*_*R*_ = 0.70; and holistic somatic stimulation, *R*_*R*_ = 0.77.

#### Study 3: Analytic Plan

The associations between pornography use, relationship satisfaction, and sexual satisfaction were analyzed using the same RSA APIM approach that was outlined in Study 1 using IBM SPSS Statistics 25 (IBM Corporation, 2017).

Erotophobia-erotophilia and each dimension of sexual preference were then used to explore statistical confounding of the associations between similarity-dissimilarity in pornography use and relationship and sexual satisfaction. To this end, we first constructed separate linear mixed APIMs for each of the six potential explanatory variables. In each case, relationship and sexual satisfaction were regressed on an actor effect, a partner effect, and the interaction between the actor and partner effects (e.g., relationship satisfaction was regressed on actors’ erotophobia-erotophilia, partners’ erotophobia-erotophilia, and the interaction between these effects). Models that produced significant interactions were assumed to be eligible candidates for testing statistical confounding with the interaction between actors’ and partners’ solitary pornography use. Actors’ and partners’ solitary pornography use and their interaction and shared pornography use were then added to these candidate models only. In all cases, ML estimation was used and residuals were modeled by nesting partner within couple using an unstructured residual covariance matrix.

### Study 3: Results

Correlations between primary measures can be found in Table 5. Strong correlations existed between men and women’s reports of relationship satisfaction, *r* = 0.77, *p* < 0.001, sexual satisfaction, *r* = 0.76, *p* < 0.001, and shared pornography use, *r* = 0.86, *p* < 0.001, as well as between the within-subject reports of sexual and relationship satisfaction provided by men, *r* = 0.77, *p* < 0.001, and women, *r* = 0.84, *p* < 0.001.

**FIGURE 4:**
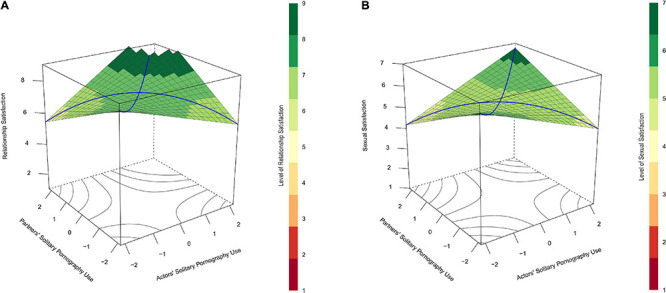
**(A)** Depicts predicted relationship satisfaction (vertical axis) as a function of actors’ (*x*-axis) and partners’ (*y*-axis) frequencies of solitary pornography use and their interaction for cases that reported mean levels of shared pornography use in Study 3. **(B)** Does the same for the prediction of sexual satisfaction. In both figures, satisfaction scores tended to be lowest in cases in which couple members were most dissimilar in their frequencies of solitary pornography use (left- and right-most corners of the plots). According to regions of significance tests, participants’ own solitary pornography use was negatively related to their relationship satisfaction if their partners’ almost never used pornography alone or never used pornography (partners’ solitary pornography use <–1.15) but was positively related to their relationship satisfaction when their partners’ used pornography alone more than once or twice a week (partners’ solitary pornography use >0.38). Similarly, further regions of significance tests found that sexual satisfaction was negatively related to participants’ solitary pornography when their partners used pornography alone less than one to three times a month (partners’ solitary pornography use <0.85) but was positively related when their partners used pornography alone more once a day (partners’ solitary pornography use >1.95).

The initial model predicted relationship satisfaction using actors’ and partners’ reports of solitary pornography use and their interaction. In subsequent steps, model fit was improved, *χ*^2^ (1) = 4.66, *p* = 0.031, by adding shared pornography use as a between-dyad covariate, and gender as a within-dyad factor, *χ*^2^ (1) = 5.13, *p* = 0.024. In this model, frequency of shared pornography use was significantly related to relationship satisfaction (H1a), *b* = 0.30, *p* = 0.03, and the positive interaction term between actors’ and partners’ solitary pornography use was significant, *b* = 0.40, *p* = 0.002 (see Table 6 and Figure 4A). These results are similar to those presented in Study 1, though in this case the interaction between actors’ and partners’ solitary pornography use remained significant after controlling for gender. The RSA of the solitary pornography use components of this model further revealed significant curves along the lines of congruence, *a*_2_ = 0.40, *p* = 0.002, and incongruence, *a*_4_ = −0.40, *p* = 0.002, which indicated a similarity-dissimilarity effect (H2a). There were no significant slopes in this analysis, *a*_1_ = 0.41, *p* = 0.236 and *a*_3_ = 0.15, *p* = 0.083, and the lack of a significant slope for *a*_3_ suggested that the effect of similarity-dissimilarity was similar regardless of who was using pornography alone in the relationship. Adding further interactions between gender and the other components of the model did not significantly improve model fit, *χ*^2^ (3) = 0.19, *p* = 0.980.

**TABLE 6:** Linear mixed models predicting relationship and sexual satisfaction for Study 3 (*N* = 207 couples).

	**Relationship Satisfaction**		**Sexual Satisfaction**	
	***s*^2^**	**p**	***s*^2^**	**p**
**Fixed Effects**				
Intercept	7.23	>0.001	5.54	>0.001
Actors’ Solitary Porn Use	0.28	0.120	0.07	0.581
Partners’ Solitary Porn Use	0.13	0.462	−0.03	0.788
Actors’ by Partners’ Solitary Porn Use	0.40	0.002	0.25	0.005
Shared Porn Use	0.30	0.031	0.45	>0.001
Gender	−0.12	0.024	−0.10	0.012

Tests for simple slopes of actors’ solitary pornography use at different values of a partners’ solitary pornography use indicated regions of significance that were less than −1.15 and greater than 0.38. These results implied that participants’ own solitary pornography use was negatively related to their relationship satisfaction if their partners’ almost never used pornography but positively related to their relationship satisfaction when their partners’ used pornography alone more than once or twice a week.

Sexual satisfaction was analyzed in the same way. In this case, adding the main effect for shared pornography use significantly improved fit, *χ*^2^ (1) = 20.76, *p* < 0.001, as did adding gender, *χ*^2^ (1) = 6.30, *p* = 0.012. As in Study 1, in this model (see Table 6 and Figure 4B), the interaction between actor’s and partners’ solitary pornography use was significant when predicting sexual satisfaction, *b* = 0.25, *p* = 0.007, as was the frequency of shared pornography use, *b* = 0.45, *p* < 0.001. The RSA of the solitary pornography use components of this model further revealed significant curves along the lines of congruence, *a*_2_ = 0.25, *p* = 0.005, and incongruence, *a*_4_ = −0.25, *p* = 0.005, however, the slopes along these lines were not significant, *a*_1_ = 0.04, *p* = 0.880, *a*_3_ = 0.10, *p* = 0.103. As with the prediction of relationship satisfaction in this sample, the significant main effect for shared pornography use indicated that couples who reported higher frequencies of shared pornography use reported higher sexual satisfaction (H1b), and the significant curve along the line of incongruence accompanied by a null slope implied the presence of similarity-dissimilarity effect in solitary pornography use that did not depend on who was using pornography alone in the relationship (H2b). Model fit was not improved by adding interactions between gender and the other components of the model, *χ*^2^ (3) = 0.33, *p* = 0.954.

Region of significance tests for simple slopes of actors’ solitary pornography use at different values of partners’ solitary pornography use revealed significant slopes outside of the region bounded by −0.85 and 1.95. These results indicated that participants’ solitary pornography use was negatively related to participants’ own sexual satisfaction when their partners used pornography less than 1–3 times per month but was positively related to their sexual satisfaction when their partners used pornography more than once a day.

#### Study 3: Similarity-Dissimilarity in Other Individual Difference and Attitudinal Dimensions

Correlations between the study’s primary measures, erotophobia-erotophilia, and each of the five sexual preference factor scores can be found in Table 7. Of particular note, participants’ preference for a loving partner was positively correlated with relationship satisfaction, *r* = 0.22, *p* < 0.001, and sexual satisfaction, *r* = 0.22, *p* < 0.001, and negatively correlated with participants’ solitary pornography use, *r* = −0.28, *p* < 0.001, and their partners’ solitary pornography use, *r* = −0.10, *p* = 0.050.

**TABLE 7:** Correlations between primary variables and potential “third variables” for Study 3 (*N* = 207 couples).

	**Relationship Satisfaction**	**Sexual Satisfaction**	**Actors’ Porn. Use**	**Partners’ Porn. Use**	**Shared Porn. Use**
**Sexual Ideals**					
Aggressive Sex	0.09	0.12*	0.26*	0.13*	0.21*
Loving Partner	0.22*	0.22*	−0.28*	−0.10*	−0.07
Pornographic Sex	0.05	0.08	0.30*	−0.03	0.21*
Partner Characteristics	0.07	0.09	−0.13*	−0.15*	−0.09
Holistic Stimulation	0.15*	0.18*	0.18*	0.04	0.28*
Erotophobia-Erotophilia	0.02	0.03	0.45*	0.13*	0.33*

*r > 0.09 is *p* < 0.05; *r* > 0.12 is *p* < 0.01; *r* > 0.16 is *p* < 0.001.

When similarity-dissimilarity models were constructed to predict relationship satisfaction using these individual difference measures, significant interactions between actor and partner effects were only found when examining erotophobia-erotophilia, *b* = 0.27, *p* = 0.014, and preference for pornographic sex, *b* = 0.27, *p* = 0.019. While both models resulted in prediction surfaces that were comparable to those found for similarity-dissimilarity in solitary pornography use, model fit was improved in both cases when the effects for solitary pornography use were added (erotophobia-erotophilia model, *χ*^2^ (4) = 27.30, *p* < 0.001; preference for pornographic sex model, *χ*^2^ (4) = 20.97, *p* < 0.001). Moreover, in these combined models, the interactions between actors’ and partners’ solitary pornography use were significant while controlling for similarity-dissimilarity in erotophobia-erotophilia, *b* = 0.40 *p* = 0.002, and preference for pornographic sex, respectively, *b* = 0.37 *p* = 0.004. On the basis of these results, it does not appear that the similarity-dissimilarity effect of actors’ and partners’ solitary pornography use on relationship satisfaction can be explained by similarity-dissimilarity in erotophobia-erotophilia or sexual ideal preferences (RQ1).

When the same approach was applied to sexual satisfaction, significant interactions between actor and partner effects were limited to similarity-dissimilarity models involving erotophobia-erotophilia, *b* = 0.26, *p* < 0.001, preference for pornographic sex, *b* = 0.30, *p* = 0.014, and preference for holistic somatic stimulation, *b* = 0.17, *p* = 0.023. Similar to the analysis of relationship satisfaction, the addition of the effects of solitary pornography use resulted in significant interactions between actors’ and partners’ solitary pornography use when erotophobia-erotophilia was controlled for, *b* = 0.23, *p* = 0.010, when preference for pornographic sex was controlled for, *b* = 0.21, *p* = 0.014, and when preference for holistic somatic stimulation was controlled for, *b* = 0.21, *p* = 0.016, and model fit was improved in all cases [erotophobia-erotophilia model, *χ*^2^ (4) = 35.13, *p* < 0.001; preference for pornographic sex model, *χ*^2^ (4) = 34.61, *p* < 0.001; preference for holistic somatic stimulation *χ*^2^ (4) = 31.70, *p* < 0.001]. Unlike the examination of relationship satisfaction, however, the magnitude of the interaction between actors’ and partners’ solitary use appeared to be marginally reduced in each case. Still, the results did not clearly support the notion that any of these variables are strongly implicated as potential explanatory mechanisms that underlie the associations between similarity-dissimilarity in solitary pornography use and relationship and sexual satisfaction (RQ1).

### Study 3: Discussion

The results of the primary analyses in Study 3 largely replicate those of Study 1. Shared pornography use was positively correlated with relationship and sexual satisfaction while associations between one’s own solitary pornography use and relationship and sexual satisfaction were contingent on a partner’s solitary pornography use. With respect to the similarity-dissimilarity effects, both indicators of relationship quality were lowest when partners were highly discrepant in their frequencies of solitary pornography use and highest when both partners either did not use pornography alone or used it at a high frequency alone. Once again, couples that were characterized by mid-frequency solitary pornography use fell somewhere between these two extremes.

Our investigation of explanatory mechanisms of this phenomenon, however, came up short. While we found similarity-dissimilarity effects for couple members’ erotophobia-erotophilia, their preferences for pornographic sex, and there preferences for holistic somatic stimulation, none of these constructs accounted wholly for the similarity-dissimilarity effects of solitary pornography use. Perhaps this was because none of these variables was highly correlated with solitary pornography use (erotophobia-erotophilia: *r* = 0.45; preference for pornographic sex: *r* = 0.30; preference for holistic somatic stimulation: *r* = 0.18). Similarities and differences in preference for pornographic sex and erotophilia, while relevant to understanding differences in relationship and sexual satisfaction across couples, may simply have been too distal from pornography use behavior to account for its effects. Given erotophobia-erotophilia’s conceptual similarity to attitudes toward pornography use (Fisher et al., 1988), these results somewhat undermine the view that similarity-dissimilarity in attitudes toward pornography use account for the effects of similarity-dissimilarity in solitary pornography use either.

## Study 4: Sexual and Relationship Satisfaction Over Time

The pattern of findings across these three studies suggests a narrative that is at odds with predominant views in this field. Despite meta-analytic findings linking pornography use to lower relationship and sexual satisfaction (Wright et al., 2017), the results of the current research provide evidence that pornography use is not necessarily associated with deficiencies in relationship functioning. Specifically, relationship and sexual satisfaction appear to be higher among those who use pornography together than those that do not. Furthermore, the negative associations between solitary pornography use and relationship and sexual satisfaction appear to be mostly limited to couples that are characterized by high discordance in solitary pornography use between couple members.

In her dissertation work, Shaw (2017) independently tested similar hypotheses in a large sample of heterosexual dyads that were re-assessed at 1-month intervals for 6 months. The results reported in that research are similar to those reported in Studies 1 and 3, though differences in the analytic models preclude close comparisons. In an effort to build a cohesive examination of the associations between pornography use and relationship and sexual satisfaction, Shaw’s (2017) data were used to test H1 and H2 using variations of the models developed in Studies 1 through 3.

On the basis of our previous findings and Shaw’s (2017) original analyses, we expected that shared pornography use would be positively related to relationship and sexual satisfaction and that actors’ and partners’ solitary pornography use would interact, such that solitary pornography use would be negatively related to relationship and sexual satisfaction among couples exhibiting dissimilarity in solitary pornography use. Although Shaw (2017) reported some gender specific coefficients that suggested differences in results by gender, these differences were often small, and were not found in many of the models that were tested. Given our previous findings, we did not expect to find significant differences by gender when the data were re-analyzed. This study also afforded an opportunity to replicate the time-based analysis conducted in Study 2 with measures of pornography use rather than attitudes toward pornography use alone.

In addition, this dataset included two variables that could be used to further examine potential explanations for similarity-dissimilarity effects of solitary pornography use (RQ1). Attitudes toward a partners’ pornography use and sex drive were both measured at baseline data collection. When these predictions were registered, we believed that similarity-dissimilarity in either or both dimensions might explain the similarity-dissimilarity effects, so we further scrutinized significant interactions between actors’ and partners’ solitary pornography use by controlling for the interaction between actors’ and partners’ attitudes toward a partner using pornography, and the interaction between actors’ and partners’ sex drive (registered materials: https://osf.io/w9m6p; data and syntax^6^).

### Study 4: Method

#### Study 4: Participants

Data for this study were drawn from a large dyadic panel of adults in sexually active relationships. Of the initial *N* = 2,214 participants who completed a baseline survey, *n* = 599 romantic partners were successfully recruited into the longitudinal panel, *n* = 529 of which were heterosexual. Detailed descriptions of the selection biases for the recruited dyads can be found in Shaw (2017). For the purposes of this study, analyses were further restricted to heterosexual dyads in which both partners provided data at one or more of the first 6 follow-up waves (*n* = 277 of 529). Couple members in the retained sample were young adults (*M* = 33.07, *SD* = 11.52), Caucasian (87.73%), non-Hispanic (95.46%), and married (50.54%), who had been in their romantic relationships for *M* = 8.17, *SD* = 9.86 years.

Compared to the participants from the *n* = 252 heterosexual dyads who were not included in this study, those who were included in this study were more likely to be white, χ^2^(1) = 15.95, *p* < 0.001, and were older, *t*(1054) = −5.82, *p* < 0.001, had more years of education, *t*(1013) = −6.18, *p* < 0.001, higher incomes, *t*(1044) = −3.12, *p* = 0.002, longer relationships, *t*(985) = −5.19, *p* < 0.001, higher relationship satisfaction, *t*(1056) = −1.96, *p* = 0.049, more positive attitudes toward a partner’s pornography use, *t*(913) = −1.97, *p* = 0.049, and lower sex drive, *t*(1054) = 2.22, *p* = 0.027. These two groups did not differ significantly in their solitary pornography use, *t*(970) = 1.78, *p* = 0.075, shared pornography use, *t*(1056) = 0.56, *p* = 0.562, sexual satisfaction, *t*(1052) = −0.14, *p* = 0.890, or sexual dissatisfaction, *t*(1049) = 0.06, *p* = 0.957.

#### Study 4: Materials and Procedure

Couples who participated in the baseline survey were sent e-mail invitations to complete 11 brief follow-up surveys at 1-month intervals and a final 12th outgoing survey that was more comprehensive in nature. The data used in this study were limited to the baseline assessment through the 6th follow-up due to increasing participant attrition. The current study made use of baseline assessments of sex drive and attitudes toward pornography use in conjunction with follow-up measures of pornography use, relationship satisfaction, sexual satisfaction, and sexual dissatisfaction. Unfortunately, operational differences between baseline and follow-up assessments of sex-drive, pornography use, and sexual dissatisfaction precluded the possibility of creating analytic models that included both baseline and successive assessments of these variables as equivalent “waves” of data. Monetary rewards and raffle opportunities for goods were used to incentivize participation, and all procedures were reviewed and approved by an institutional review board before data collection began. Further details concerning the procedure and other measures can be found in https://osf.io/w9m6p. Means and standard deviations for the following measures can be found in Table 8.

**TABLE 8:** Summary of the correlations, means, and standard deviations of the focal variables for Study 4 (*N* = 277 couples).

	**1**	**2**	**3**	**4**	**5**	**6**	**7**	**8**	**9**	**10**	***M***	***SD***
1. Relationship Satisfaction Male											4.76^1^	1.01
2. Relationship Satisfaction Female	0.58**										4.97	0.90
3. Sexual Satisfaction Male	0.63**	0.38**									3.97	1.59
4. Sexual Satisfaction Female	0.35**	0.47**	0.34**								4.19	1.50
5. Sexual Dissatisfaction Male	−0.52**	−0.41**	−0.67**	−0.20**							1.61	1.06
6. Sexual Dissatisfaction Female	−0.31**	0.43**	−0.32**	−0.62**	0.28**						1.44	0.82
7. Solitary Porn. Use Male	–0.03	–0.06	−0.19**	–0.10	0.17*	0.08					1.36^1^	1.27
8. Solitary Porn. Use Female	–0.03	0.00	0.00	–0.11	0.10	0.14*	0.05				0.40	0.89
9. Shared Porn. Use Male	0.13	0.12	0.15*	0.14*	−0.14*	–0.09	0.12*	0.05			0.21	0.51
10. Shared Porn. Use Female	0.11	0.11	0.14*	0.20**	–0.04	–0.15	0.08	0.25**	0.58**		0.18	0.48
11. Mean Shared Porn. Use	0.14*	0.14*	0.17**	0.20**	–0.11	–0.14	0.11	0.18*	0.90**	0.88**	0.20	0.44

With respect to relationship satisfaction, sexual satisfaction, and sexual dissatisfaction, only correlations with these measures at the first assessment are presented.

*Signifies significant correlations *p* < 0.05; **signifies significant correlations, *p* < 0.01.

*^1^Men report significantly lower relationship satisfaction, *p* < 0.001, and higher solitary pornography use, *p* < 0.001, than women.*

*For interpretability, all *M* and *SD* are presented in the original scale metric.*

##### Study 4: relationship satisfaction

Relationship satisfaction was measured with the four item Couples Satisfaction Index (Funk and Rogge, 2007) at all six waves. Item descriptions can be found in Study 1. Responses were summed so that higher scores reflected higher levels of relationship satisfaction (Cronbach’s α = 0.90 – 94).

##### Study 4: sexual satisfaction

Sexual satisfaction and sexual dissatisfaction were measured separately using two items each from the Quality of Sex Inventory (Shaw and Rogge, 2016) at each of the six waves. Specifically, sexual satisfaction was assessed with “My sex life is fulfilling” and “I am satisfied with our sexual relationship” while sexual dissatisfaction was assessed with “Sexual activity with my partner was not fun” and “I was very disappointed with my sex life with my partner.” Responses were collected on 6-point scales that ranged from 1 “Not at all TRUE” to 6 “Completely TRUE” and were mean aggregated so that higher scores reflected higher levels of sexual satisfaction and dissatisfaction, respectively. These scales demonstrated high internal consistency in the current sample (sexual satisfaction: α = 0.93 –0.97; sexual dissatisfaction: α = 0.79 – 88).

##### Study 4: pornography use

At each of 6 waves following baseline assessment, participants read the stem, “IN THE LAST WEEK, how often did you and your partner view erotic material or engage in sexually charged experiences (visiting/viewing websites, chat rooms, magazines, or movies with adult content, or going to strip clubs or live shows)” and responded to following two items: “How often did you do any of these things WITHOUT your partner?” and “How often did you do any of these things WITH your partner?”. Responses were collected with an 8-point scale (*0 times* to *13+ times*). Responses were averaged across waves to create single time invariant estimates of solitary and shared pornography use for each participant in this study (rationale described in Supplementary Data Sheet 2). As with Studies 1 and 3, non-use of pornography was common and responses were positively skewed in this sample: 40.43% of the sample reported never using pornography alone (*S* = 2.03, *p* < 0.001) while 64.62% reported never using it with a partner (*S* = 4.71, *p* < 0.001). Reports of shared pornography use by each partner were moderately correlated, *r* = 0.58, *p* < 0.001, and were mean averaged to create a time invariant dyadic index of shared pornography use. All measures of pornography use were standardized and re-centered at the midpoint of the scale range for use in the RSAs described below.

##### Study 4: sex drive

Four items were used to assess participants’ sex drives at baseline: “I find myself craving sex often”; “I tend to be horny most of the time”; “My mind often wanders to sex”; and “I can get turned on very quickly.” These items were rated on 5-point response scales that ranged from 1 “Not at all TRUE” to 5 “Very TRUE” and were mean averaged so that higher scores reflected higher sex drive (α = 0.92). Aggregate scores were then standardized for use in the analyses described below.

##### Study 4: attitudes toward a partner’s pornography use

Following baseline measures of pornography use, one item assessed attitudes toward a partners’ pornography use: “How upset are YOU over your partner engaging in these activities? (if your partner engages in them at all)” Responses were collected in a 6-point scale that ranged from 1 “Not at all” to 6 “Completely.” This item was reverse scored and responses were standardized for use in the analyses described below.

#### Study 4: Analytic Plan

We departed from our pre-registered analytic plan involving time varying estimates of pornography use after executing it because we realized that weekly frequency of pornography use measures failed to identify many female pornography users (for the full rationale, see Appendix B). Despite some modifications, the resulting analytic approach followed the same general plan outlined in the pre-registered analyses. The initial linear mixed modeling approach involved the prediction of each of three time varying dependent variables (relationship satisfaction, sexual satisfaction, and sexual dissatisfaction) with time invariant actors’ solitary pornography use, partners’ solitary pornography use, their interaction, and shared pornography use. Following Kenny et al.’s (2006) recommendations for repeated measures APIMs, residuals were modeled by crossing partner with time nested within couple and constraining the resulting covariance matrix to a heterogeneous autoregressive structure. ML estimation was used so that nested models could be tested for changes in model fit. Subsequent models tested the addition of gender and interactions between pornography components and gender, as well as time, and interactions between pornography components and time.

When significant interactions between actors’ and partners’ solitary pornography use emerged, two further fixed effect models were considered to examine possible explanations for these effects. First, the basic fixed effects model with all pornography use components but without gender or time components was re-run with the addition of fixed effects for actors’ sex drive, partners’ sex drive and the interaction between actors’ and partners’ sex drive. The second model was similar, but replaced sex drive with attitudes toward a partner’s use of pornography.

### Study 4: Results

Correlations between primary measures can be found in Table 8. At the first wave, a moderate correlation existed between men’s and women’s reports of relationships satisfaction, *r* = 0.58, *p* < 0.001, but correlations were weaker for sexual satisfaction, *r* = 0.34, *p* < 0.001 and sexual dissatisfaction, *r* = 0.28, *p* < 0.001. The correlation between partners’ reports of solitary pornography use, *r* = 0.05, *p* = 0.376, was also much lower than expected.

**FIGURE 5:**
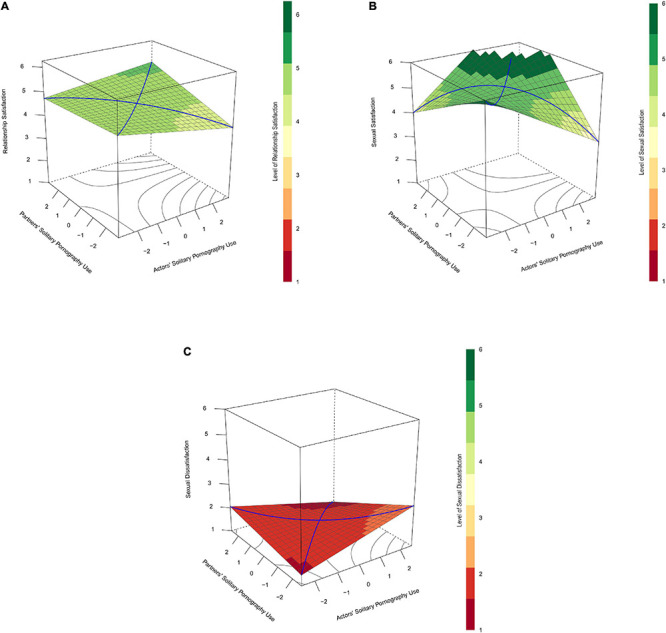
**(A)** Depicts predicted relationship satisfaction (vertical axis) as a function of actors’ (*x*-axis) and partners’ (*y*-axis) frequencies of solitary pornography use and their interaction for cases that reported mean levels of shared pornography use in Study 4. **(B,C)** Do the same for the prediction of sexual satisfaction and sexual dissatisfaction, respectively. In **(A)**, the lack of significant interaction resulted in a flatter prediction surface where partners with dissimilar frequencies of solitary pornography use were not notably lower in relationship satisfaction than partners with more similar frequencies. In **(B)**, sexual satisfaction scores tended to be lowest in cases in which couple members were most dissimilar in their frequencies of solitary pornography use (left- and right-most corners of the plots). According to regions of significance tests, participants’ own solitary pornography use was negatively related to their relationship satisfaction if their partners’ used pornography alone less than 1.80 times a week but was otherwise unrelated. Conversely, in **(C)**, sexual dissatisfaction scores tended to be highest in cases in which couple members were most dissimilar in their frequencies of solitary pornography use (left- and right-most corners of the plots). According to regions of significance tests, participants’ solitary pornography use was positively related sexual dissatisfaction if their partner indicated that they used pornography less than 2.17 times a week, otherwise their pornography use was unrelated to their sexual dissatisfaction.

In the first step, relationship satisfaction was predicted with actors’ and partners’ reports of solitary pornography use, their interaction, and their reports of shared pornography use. This model indicated a significant positive main effect for shared pornography use, *b* = 0.13, *p* = 0.002, but the interaction between actors’ and partners’ pornography use was not significant, *b* = 0.04, *p* = 0.395 (see Table 9 and Figure 5A). As with Study 1, such results support the view that relationship satisfaction was higher among those who shared pornography use more frequently (H1a) but not among those who were more similar in their solitary pornography use (H2a). Adding gender and further interactions between gender and the other components of the model did not significantly improve model fit, *χ*^2^ (5) = 2.65, *p* = 0.754, nor did adding time components, *χ*^2^ (5) = 4.71, *p* = 0.452.

**TABLE 9:** Linear mixed models predicting relationship and sexual satisfaction for Study 4 (*N* = 277 couples).

	**Relationship Satisfaction**		**Sexual Satisfaction**		**Sexual Dissatisfaction**	
	***b***	***p***	***b***	***p***	***b***	***p***
**Fixed Effects**						
Intercept	4.75	> 0.001	3.85	> 0.001	1.78	> 0.001
Actors’ Solitary Porn Use	−0.03	0.821	0.09	0.614	0.04	0.731
Partners’ Solitary Porn Use	0.09	0.493	0.23	0.191	−0.24	0.042
Actors’ by Partners’ Solitary Porn Use	0.04	0.395	0.19	0.006	−0.14	0.003
Shared Porn Use	0.13	0.002	0.34	> 0.001	−0.17	>0.001
Gender	−	−	−	−	−0.51	0.007
Gender by Actor’s Porn Use	−	−	−	−	−0.20	0.006
Time	−	−	−0.07	> 0.001	0.03	0.031

Sexual satisfaction was analyzed in the same way. The initial model, which predicted sexual satisfaction using actors’ and partners’ reports of solitary pornography use, their interaction, and their reports of shared pornography use, revealed a significant main effect for shared pornography use, *b* = 0.34, *p* < 0.001, and a significant positive interaction between actors’ and partners’ solitary pornography use, *b* = 0.19, *p* = 0.008 (see Figure 5B). The RSA of this model further revealed significant curves along the lines of congruence, *a*_2_ = 0.19, *p* < 0.009, and incongruence, *a*_4_ = −0.19, *p* < 0.009, and a significant slope along the line of incongruence *a*_3_ = −0.12, *p* = 0.043 but not along the line of congruence *a*_1_ = 0.30, *p* = 0.401. The *a*_3_ slope suggested that the dissimilarity effect in sexual satisfaction was more pronounced among the partners who use pornography alone than the partners that do not. These effects again confirmed that sexual satisfaction was higher among participants who reported more shared pornography use (H1b) and more similar solitary pornography use in this sample (H2b).

Region of significance tests indicated significant slopes for actors’ solitary pornography use when partners’ solitary pornography use was less than −1.40. The slopes for actors’ solitary pornography use were not significant above this point. These results suggested that participants’ solitary pornography use was negatively related to sexual satisfaction if their partners’ used pornography less frequently than 1.80 times a week but was otherwise unrelated.

Adding further interactions between gender and the other components of the model did not significantly improve model fit, *χ*^2^ (5) = 2.92, *p* = 0.712. Adding a main effect for time significantly improved model fit, *χ*^2^ (1) = 15.79, *p* < 0.001, resulting in a significant negative main effect for time, *b* = 0.34, *p* < 0.001, which indicated that sexual satisfaction generally decreased over the course of the study (see Table 9). Adding additional interactions between time and other components of the model did not improve fit further, *χ*^2^ (4) = 3.39, *p* = 0.495. This negative main effect for time did not diminish the significant interaction between actors’ and partners’ solitary pornography use appreciably.

Analysis of sexual dissatisfaction followed the same approach, beginning with a fixed effects model that included actors’ and partners’ solitary pornography use, their interaction, and shared pornography use. As expected, there was a negative main effect for shared pornography use, *b* = −0.17, *p* < 0.001, and a negative interaction between actors’ and partners’ solitary pornography use, *b* = −0.09, *p* = 0.042 (see Figure 5C). The RSA of this model further revealed significant curves along the lines of congruence, *a*_2_ = −0.09, *p* = 0.043, and incongruence, *a*_4_ = 0.09, *p* = 0.043, and a significant slope along the line of incongruence *a*_3_ = 0.12, *p* = 0.001, but not along the line of congruence *a*_1_ = −0.09, *p* = 0.673. As with the analysis of sexual satisfaction, this significant slope indicated that the effects of dissimilarity in solitary pornography use were particularly prominent among the pornography user rather than the non-user. In this case, participants who reported more sexual dissatisfaction tended to report lower frequencies of shared pornography use (H1b), and more dissimilar frequencies of solitary pornography use (H2b).

Region of significance tests indicated significant slopes for actors’ solitary pornography use when partners’ solitary pornography use was less than −1.18 and slopes above this point were not significant. These results suggested that participants’ solitary pornography use was positively related to their sexual dissatisfaction if their partner indicated that they used pornography less frequently than 2.17 times a week, otherwise their pornography us was unrelated to their sexual dissatisfaction.

Adding gender and an interaction between gender and actors’ solitary pornography use improved fit, *χ*^2^ (2) = 7.29, *p* = 0.019. In this model, gender interacted with actors’ solitary pornography use such that being male reduced the association between actors’ solitary pornography use and sexual dissatisfaction, *b* = −0.20, *p* = 0.006, a rather surprising finding. Although this was not of particular interest in the current study, it appeared that in the context of this particular model, on average, men’s solitary pornography use was related to lower sexual dissatisfaction while the same was not true of women. Adding further interactions with gender did not significantly improve fit, *χ*^2^ (3) = 1.34, *p* = 0.720. However, fit was improved when a fixed effect for time was added, *χ*^2^ (1) = 4.61, *p* = 0.032, because sexual dissatisfaction appears to have increased over time, *b* = −0.03, *p* = 0.031. Adding additional interactions between time and pornography use did not improve fit, *χ*^2^ (4) = 2.84, *p* = 0.585. The significant interaction between actors’ and partners’ solitary pornography remained when controlling for these additional components (see Table 9).

#### Testing Explanations for the Interaction When Predicting Sexual Satisfaction

To test the possible influence of similarity-dissimilarity in sex drive (RQ1), actors’ baseline sex drive, partners’ baseline sex drive, and the interaction between actors’ and partners’ baseline sex drive were added to the model predicting sexual satisfaction without gender or time components. These additions significantly improved fit, *χ*^2^ (3) = 18.36, *p* < 0.001, and in the resulting model the main effect for shared pornography use remained significant, *b* = 0.27, *p* < 0.001, and the interaction between actors’ and partners’ solitary pornography use dropped to non-significance, *b* = 0.14, *p* = 0.062 (see Table 10). These results suggest that partner similarity-dissimilarity in sex drive may be linked to the association between similarity-dissimilarity in solitary pornography use and sexual satisfaction.

**TABLE 10:** Testing potential confounding effect of differences in actors’ and partners’ attitudes toward pornography in Study 4 (*N* = 233 couples).

	**Sexual Satisfaction**				**Sexual Dissatisfaction**			
	**Base Model**		**Sex Drive Model**		**Base Model**		**Sex Drive Model**	
	***b***	***p***	***b***	***p***	***b***	***p***	***b***	***p***
**Fixed Effects**								
Intercept	3.68	> 0.001	3.32	> 0.001	1.77	> 0.001	1.92	> 0.001
Actors’ Solitary Porn Use	0.09	0.612	−0.03	0.826	0.02	0.891	0.09	0.434
Partners’ Solitary Porn Use	0.21	0.248	0.04	0.786	−0.11	0.337	−0.02	0.881
Actors’ by Partners’ Solitary Porn Use	0.19	0.008	0.13	0.062	−0.09	0.042	−0.05	0.262
Shared Porn Use	0.34	> 0.001	0.27	> 0.001	−0.17	> 0.001	−0.14	> 0.001
Actors’ Sex Drive	−	−	0.19	> 0.001	−	−	−0.03	0.368
Partners’ Sex Drive	−	−	0.25	> 0.001	−	−	−0.07	0.027
Actors’ by Partners’ Sex Drive	−	−	0.09	0.072	−	−	−0.12	> 0.001

The influence of similarity-dissimilarity in attitudes toward a partners’ use of pornography (RQ1) was examined by adding actors’ and partners’ baseline attitudes toward a partner’s use of pornography and their interaction to the model without time. These additions significantly, improved fit, *χ*^2^ (3) = 9.73, *p* = 0.021, and in the resulting model, both the main effect for shared pornography use, *b* = 0.30, *p* < 0.001, and the interaction between actors’ and partners’ solitary pornography use, *b* = 0.17, *p* = 0.016, remained significant and largely unperturbed (see Table 11). With respect to the attitudinal components themselves, participants who had more positive attitudes toward their partners’ pornography use reported higher levels of sexual satisfaction than participants with more negative attitudes, but partners’ attitudes, and the interaction between actors’ and partners’ attitudes appeared to be unrelated.

**TABLE 11:** Testing potential confounding effect of differences in actors’ and partners’ attitudes toward pornography in Study 4 (*N* = 233 couples).

	**Sexual Satisfaction**				**Sexual Dissatisfaction**			
	**Base Model**		**Sex Drive Model**		**Base Model**		**Sex Drive Model**	
	***b***	***p***	***b***	***p***	***b***	***p***	***b***	***p***
**Fixed Effects**								
Intercept	3.37	> 0.001	3.65	> 0.001	1.78	> 0.001	1.66	> 0.001
Actors’ Solitary Porn Use	0.03	0.884	0.06	0.748	0.04	0.732	−0.01	0.916
Partners’ Solitary Porn Use	0.15	0.401	0.19	0.285	−0.10	0.375	−0.16	0.152
Actors’ by Partners’ Solitary Porn Use	0.16	0.028	0.17	0.016	−0.08	0.066	−0.10	0.019
Shared Porn Use	0.32	> 0.001	0.30	> 0.001	−0.16	> 0.001	−0.14	> 0.001
Actors’ Attit. Toward Porn Use	−	−	0.17	0.004	−	−	−0.16	> 0.001
Partners’ Attit. Toward Porn Use	−	−	0.09	0.127	−	−	−0.08	0.035
Actors’ by Partners’ Attit. Toward Porn Use	−	−	0.05	0.123	−	−	−0.06	0.003

#### Testing Explanations for the Interaction When Predicting Sexual Dissatisfaction

To test the influence of similarity-dissimilarity in sex drive (RQ1), actors’ baseline sex drive, partners’ baseline sex drive, and the interaction between actors’ and partners’ baseline sex drive were added to the base model predicting sexual dissatisfaction without gender or time components. These additions significantly improved fit, *χ*^2^ (3) = 20.29, *p* < 0.001, and in the resulting model, the main effect for shared pornography use remained significant, *b* = −0.14, *p* < 0.001, and the interaction between actors’ and partners’ solitary pornography use dropped to non-significance, *b* = −0.05, *p* = 0.262 (see Table 10). Such results suggest that similarity-dissimilarity in sex-drive may be implicated in the association between similarity-dissimilarity in solitary pornography use and sexual dissatisfaction.

The influence of similarity-dissimilarity in attitudes toward a partner’s use of pornography was examined next^7^ (RQ1), with the addition of actors’ and partners’ baseline attitudes toward their partner’s use of pornography and the interaction to the base model without gender or time components. These additions significantly improved fit, *χ*^2^ (3) = 22.27, *p* = 0.021, and in the resulting model both the main effect for shared pornography use, *b* = −0.14, *p* < 0.001, and the interaction between actors’ and partners’ solitary pornography use, *b* = −0.10, *p* = 0.019, were significant (see Table 11). Unlike the results for sexual satisfaction, this model resulted in a significant negative interaction between actors’ and partners’ attitudes toward pornography, *b* = −0.06, *p* = 0.003, suggesting a similarity-dissimilarity effect on sexual dissatisfaction. Controlling for this effect, however, did not eliminate the similarity-dissimilarity effect for solitary pornography use.

### Study 4: Discussion

Similar to the findings presented in Studies 1 and 3, the results of Study 4 indicated that shared pornography use was related to higher relationship and sexual satisfaction, and lower sexual dissatisfaction. As in Study 1, clear similarity-dissimilarity effects of solitary pornography use were not found when examining relationship satisfaction, though the expected interaction emerged when examining sexual satisfaction and dissatisfaction. In this case, sexual satisfaction was lowest (and sexual dissatisfaction was highest), among couples that were discordant in their solitary pornography use, particularly among the relationship partner who used pornography frequently.

The results of Study 4 are also the first to indicate overlap between the similarity-dissimilarity effect of solitary pornography use and partner similarity-dissimilarity in one of the proposed explanatory variables. Specifically, when analyzing sexual satisfaction and dissatisfaction, the interaction between partners’ sex drives but not the interaction between their attitudes toward their partner’s use of pornography, was partially confounded with interaction between actors’ and partners’ solitary pornography use, reducing this effect to non-significance. These findings are consistent with the possibility that the concordance-discordance effects of solitary pornography use may be an extension of, or contribute to, partner similarity-dissimilarity in sex drive.

## General Discussion

Building on previous research indicating positive correlations between pornography use and relationship functioning (Kohut et al., 2017b, 2018), the current work sought to determine if associations between pornography use and relationship and sexual satisfaction may vary as a function of different dyadic patterns of pornography use within adult relationships. Across three studies, we found consistent evidence that partners who watch pornography together report higher relationship and sexual satisfaction than partners who do not, and notably, this association was not moderated by gender. Independent of this association, we also found evidence of a similarity-dissimilarity effect, such that the solitary pornography use of one partner was negatively associated with their own relationship and sexual satisfaction, but only in cases where their romantic partners used little or no pornography alone^8^. Further, satisfaction measures tended to be highest among couples in which both partners either used pornography at a high frequency or did not use pornography at all. In probing potential mechanisms for the similarity-dissimilarity effect, we found that similarity-dissimilarity in sex drive, but not attitudes toward pornography, erotophobia-erotophilia, or sexual preferences may be implicated.

The most robust finding in the current analysis was that the frequency of shared pornography use was positively associated with both relationship and sexual satisfaction. These findings corroborate previous reports of similar associations in research that failed to control for similarity-dissimilarity in partners’ solitary pornography use (Bridges and Morokoff, 2011; Maddox et al., 2011; Willoughby and Leonhardt, 2020), and extend Kohut et al.’s (2018) findings that shared pornography use is associated with more open sexual communication and higher interpersonal closeness. Positive associations between shared pornography use and relationship functioning are difficult to explain with harm-focused exposure-based paradigms that draw heavily from objectification, social comparison, and script theories. Such findings, however, are quite consistent with descriptions of shared pornography use as a novel and exciting couples’ activity (Kohut et al., 2017b), as well as more general theories and evidence that link the experience of shared novel and exciting activities with relationship functioning (Aron et al., 1992, 2000; Reissman et al., 1993). Further experimental research in this vein should consider whether the introduction of (or increase in) shared pornography use can improve relationship and sexual satisfaction within couples to determine if causal claims are warranted.

While the results were less robust, it is more intriguing that similarity-dissimilarity in solitary pornography use was associated with sexual satisfaction and, to a lesser extent, relationship satisfaction. Across Studies 1, 3, and 4, we found consistent evidence indicating that the well-established negative association between pornography and sexual satisfaction was limited to cases where partners were very dissimilar in their solitary pornography use. We also found evidence that solitary pornography use was positively related to sexual and relationship satisfaction among couples in which both members frequently used pornography alone, but such effects were limited to Study 3. When considering these findings in conjunction with past research (Kohut et al., 2018), we are inclined to believe that the positive associations between solitary pornography use and relationship quality reported in Study 3 were a result of chance variation and will be unlikely to replicate in future research. Moreover, it is evident to us that dissimilarity in solitary pornography use is much more common than similarity in moderate to frequent solitary pornography use (Kohut et al., 2017a), at least with respect to the heterosexual couples that have been studied. Consequently, we are left to conclude that while solitary pornography use may typically be associated with poor relationship functioning within most heterosexual romantic couples (Wright et al., 2017), there exist at least some cases where it is not. With respect to Holbert and Park’s (2020) classification of interaction types, the interaction between heterosexual couple members’ solitary pornography use would best be described as a form of contingent moderation with a divergent negative pattern.

Such findings are nevertheless important for a number of reasons. First, if one takes the position that pornography causes relationships to deteriorate then these findings indicate important boundary conditions that limit pornography’s harmful effects to relationships with particular patterns of dissimilar pornography use. Second, these results accord nicely with well-established findings that similarity-dissimilarity in attitudes, personality, and sexual preferences are related to enhanced attraction and relationship functioning (Smith et al., 1993; Purnine and Carey, 1999; Montoya and Horton, 2013), which implies that mechanisms that are not premised on the impact of exposure to sexual content may be responsible for at least some of the purported “harms” of pornography. Finally, the lack of evidence indicating that the similarity-dissimilarity effects were further moderated by gender reinforces the possibility that previously reported gender differences in the associations between pornography use and relationship functioning (Wright et al., 2017) actually represent similarity-dissimilarity effects, rather than gender-specific responses to sexual media. While intriguing, this last speculation can only be tested conclusively with large dyadic samples of male and female same-sex relationships. Nevertheless, the current results call into question the utility of further theorizing about male- and female-specific relationship “consequences” of exposure to sexual media until such research can be conducted.

Our efforts to probe potential mechanisms for the associations between similarity-dissimilarity in solitary pornography use and relationship and sexual satisfaction corroborated previous reports that similarity-dissimilarity in attitudes (Montoya and Horton, 2013), erotophobia-erotophilia (Smith et al., 1993), sexual preferences (Purnine and Carey, 1999), and sex drive (Davies et al., 1999; Mark, 2015) are related to relationship functioning. Of particular relevance to the current analysis, similarity-dissimilarity in sex drive, but not attitudes toward one’s own pornography use, attitudes toward a partner’s pornography use, erotophobia-erotophilia, or sexual preferences, statistically accounted for similarity-dissimilarity effects of solitary pornography use. Specifically, in Study 4, once couple differences in similarity-dissimilarity in sex drive were controlled for, patterns of solitary pornography use within couples were unrelated to their sexual satisfaction. In this case, neither similarity-dissimilarity in pornography use nor sex-drive “dominated” the statistical model as such associations effectively canceled each other out. Independent of the issue of similarity-dissimilarity, both partners’ levels of sex drive in this model, but not their levels of solitary pornography use, were positively associated with sexual satisfaction. This suggests the presence of connections between sex-drive and sexual satisfaction, that are independent of solitary pornography use. It is also notable that controlling for similarity-dissimilarity in sex-drive did not interfere with the association between shared pornography use and sexual satisfaction. We believe that this latter finding reinforces the notion that the relationship correlates of shared pornography use and similarity-dissimilarity in solitary pornography use operate through different causal pathways.

The statistical overlap between similarity-dissimilarity in solitary pornography use and sex drive may be especially notable because similarity-dissimilarity in solitary pornography use was more reliably connected to sexual rather than relationship satisfaction, despite the high correlations between these two constructs. In this connection it is also worth noting that past research has indicated that pornography use has a modestly stronger association with sexual satisfaction than relationship satisfaction (Wright et al., 2017). While very speculative, such findings coupled with our own incline us to believe that at least some of the association between pornography use and relationship satisfaction may be a downstream consequence of a more proximal relationship between pornography use and sexual satisfaction, rather than vice versa. If that is the case then ameliorating sexual dissatisfaction among couples who are dissimilar in solitary pornography use by directly addressing their sexual concerns related to pornography or by tackling factors like dissimilarity in sex drive might have further salutary effects on other aspects of their relationship quality (e.g., relationship satisfaction, interpersonal closeness, commitment, etc.).

The exact nature of the relationship between solitary pornography use and sexual satisfaction remains an open question. The ACE perspective, with its emphasis on antecedent conditions and potentially spurious associations, would suggest that partner discrepancies in sex drive – which are common in heterosexual relationships (Ellison, 2002) – may precipitate and maintain dissimilarities in solitary pornography use, and potentially independent from that, fuel sexual dissatisfaction in relationships. In other words, the similarity-dissimilarity effects of solitary pornography use may have little or no impact on sexual satisfaction and may simply represent a “marker” of the causal relationship between dissimilarity in sex-drive and sexual satisfaction. However, other views would stress the possibility that our findings represent evidence that sex drive mediates the relationship between pornography use and sexual satisfaction (e.g., Wright, 2021b). That is, solitary pornography use may fundamentally increase users’ sex drives, creating imbalances in desire in the relationship, which ultimately lead to decreased sexual satisfaction for both partners. The results of Study 4 are equally consistent with both possibilities, though we would caution somewhat against the latter view. Pornography clearly induces sexual arousal in many people, but compelling data concerning pornography-induced long-term changes in people’s general levels of sexual desire are scarce. The only relevant data that we are aware of indicates that perceived increases in sex drive stemming from pornography use are not particularly common and are about equally balanced by reports that pornography use decreases sexual interests (Grov et al., 2011; Kohut et al., 2017b). Regardless, assuming our pattern of findings with respect to sex-drive are robust and replicate, further work seeking to understand the role of sex drive in the associations between solitary pornography use and sexual satisfaction will need to consider experimental designs that attempt to manipulate both sex drive and solitary pornography use independently and follow couples over time.

### Limitations

As is typically the case, the implications of this work are constrained by several important limitations. First, while we have speculated about several potential causal paths that could explain the associations between pornography use and relationship quality, these possibilities cannot be adequately tested with the current studies. We would also like to note that while our causal speculations are premised in part on research involving the experimental manipulation of perceived similarity and the introduction of shared novel activities among couples, we are quite open to the possibility that we are wrong, and other causal arrangements of the relevant constructs provide better explanations. Second, although one of these studies employed a quota sampling approach to approximately match the distribution of age and political affiliation of married American women, the remaining studies relied on convenience samples of Americans, limiting the generalizability of the current findings. Third, none of the current studies was expressly designed to examine the hypotheses of interest. Had they been, design elements, particularly the inclusion and operationalization of specific measures, would have been more consistent across studies. Relatedly, the particular operationalizations of pornography use employed in these studies may be suspect. The measure employed in Study 4 was conceptually broader than the measures used in Study’s 1 and 3 as it included “sexually charged” situations like visiting a strip-club and sex chatting, which are explicitly excluded in the other studies. While this is a poor defense, their currently exists no thoroughly validated measure of pornography use, nor any consensus on the best conceptual and operational definitions of this construct (Short et al., 2012; Kohut, 2014; Kohut et al., 2020). Given both the single-item assessments of pornography use and their different operationalizations across studies, it is at least promising that similar patterns of results emerged across our studies. Finally, while we made efforts to register all analytic plans before conducting the analyses, only Study 3 pre-registered these analyses before the data had been examined in any respect. In all other occasions, we had indications that similarity-dissimilarity effects for solitary pornography use emerged when different, yet closely related variables or models were tested. As a consequence, we would recommend that readers interpret the results of Studies 1, 2, and 4 as corroborative exploratory evidence for a pattern of results we confirmed in Study 3.

### Conclusion

In recent years, many communities, particularly in North America, have been entertaining notions that pornography constitutes a “public health crisis” (Nelson and Rothman, 2020) in part because of its purported effects on romantic relationships. This contemporary moral panic (Barnett, 2020) is driven by the conjoint efforts of radical feminist scholars and activists (Dines, 2016) and conservative religious organizations (Hamblin, 2016). Such individuals rely heavily on research that offers exposure-based explanations of study findings to justify their assertions of harm (see expert testimony provided to the Canadian Parlimentary Committee on Health, Mulley, 2017). It should be clear from our review of the literature, and the nature of the results across our studies, that an exposure-based explanation of the association between pornography use and poor relationship quality is only one of various potential mechanisms that may be at play. The current findings highlight how our collective understanding of the impact of pornography on relationships is still developing. These issues are very complex, and it seems unlikely to us that useful explanations will eventually boil down to popular epithets like “Porn Kills Love!”. It is our hope that this research will help our field move beyond simple “monkey see, monkey screw” explanations of pornography’s impact by incorporating more thorough considerations of the context of pornography use within relationships and the antecedents of such use (Campbell and Kohut, 2017; Leonhardt et al., 2019; Willoughby et al., 2020), as well as the panoply of known correlates and confounding variables (Baer et al., 2015; Perry, 2019; Vaillancourt-Morel et al., 2019; Fisher and Kohut, 2020; Kohut et al., 2020).

## Data Availability Statement

Publicly available datasets were analyzed in this study. This data can be found here: Open Science Framework. Study 1: https://osf.io/unf74/?view_only=fcbe67be7a0142d591a9bb87dcc994b0; Study 2: https://osf.io/65 2jg/?view_only=086e2336279f4f70bf6477e3f20503c8; Study 3: https://osf.io/4tbxu/?view_only=44b673a4ede14d31b6be29c033 eabdfe; Study 4: https://osf.io/8e9xb/?view_only=c888f7b084434 598901af4bc01c48a7a.

## Ethics Statement

The studies involving human participants were reviewed and approved by Western University, University of Rochester, University of Florida. Written informed consent for participation was not required for this study in accordance with the national legislation and the institutional requirements.

## Author Contributions

TK registered all studies, conducted all primary analyses across all four studies, and took lead on composing this manuscript. All authors provided feedback on the manuscript at various stages of development. TK, WF, and LC share responsibility for designing Study 1. JM and VR share responsibility for designing Study 2. KD, RB, and TK share responsibility for designing Study 3. AS under the supervision of RDR designed Study 4.

## Conflict of Interest

The authors declare that the research was conducted in the absence of any commercial or financial relationships that could be construed as a potential conflict of interest.

## Publisher’s Note

All claims expressed in this article are solely those of the authors and do not necessarily represent those of their affiliated organizations, or those of the publisher, the editors and the reviewers. Any product that may be evaluated in this article, or claim that may be made by its manufacturer, is not guaranteed or endorsed by the publisher.

## Footnotes

1 Note that while these examples are framed as antecedent factors for the purpose of illustration, these same factors could also be influenced by pornography use and thus be framed as effects in the ACE model. In that case, such consequents may “feed-forward,” setting subsequent antecedent conditions that encourage pornography use (or non-use) in the future.

2
https://osf.io/unf74/?view_only=fcbe67be7a0142d591a9bb87dcc994b0

3
https://osf.io/652jg/?view_only=086e2336279f4f70bf6477e3f20503c8

4 Initially, relationship satisfaction was omitted from our registered rationale and analytic plan for this study because of a miscommunication between the authors. Once this error was discovered by the first author, it was analyzed following the same plan that was employed to examine models of sexual satisfaction, detailed below.

5
https://osf.io/4tbxu/?view_only=44b673a4ede14d31b6be29c033eabdfe

6
https://osf.io/8e9xb/?view_only=c888f7b084434598901af4bc01c48a7a

7 Note, there was a reduction in sample size from *n* = 277 couples to *n* = 233 in this analysis due to missing data on the attitudinal items. For comparison purposes, the base sexual satisfaction and sexual dissatisfaction models were re-run with the reduced sample and the results were described in Table 11.

8 A reviewer expressed concern over the skewed nature of the independent and dependent variables, so we conducted additional unplanned analyses. For this purpose, models containing main effects and the interaction for actors’ and partners’ solitary pornography use and the main effect for shared use were used to predict relationship and sexual satisfaction in Studies 1, 3, and 4. These models were tested with a generalized linear mixed model approach to skewed data involving a gamma distribution and log-link function. The results of these analyses also support the inferences we make here.
