# Supplementary material for: But What’s Your Partner Up to? Associations Between Relationship Quality and Pornography Use Depend on Contextual Patterns of Use Within the Couple
Source: Front Psychol. 2021 Jul 30;12:661347. doi: 10.3389/fpsyg.2021.661347 (PMC8362880; doi:10.3389/fpsyg.2021.661347)
Supplement: Supplementary file 1 [file Data_Sheet_1.docx]

# Supplement 1

What follows is a description of ancillary analyses that were left out of the body of the manuscript for brevity. In each of the studies we further sought to explore the specific nature of the relationship of similarity-dissimilarity in pornography use and couple sexual and relationship satisfaction. Specifically, Kohut et al. (2018) unexpectedly found evidence of a specific saddle-shaped form association such that openness in sexual communication and closeness were highest among couple members who did *not* use pornography alone or who *both* used it alone very frequently. Couple openness of sexual communication and closeness were lower among couple members who both used pornography at a moderate frequency, and were lowest when one partner used pornography alone more frequently than the other. Statistically, this pattern of results is indicated by a polynomial regression equation in which the interaction term for the actor and partner components of the model is significant but quadratic actor and partner components are not. This form of concordance was not readily explainable, and may have been the result of chance variation. As an unanticipated finding we believed that it should be scrutinized further.

## Study 1

After gender was tested for inclusion in the models, quadratic effects for actors’ and partners’ solitary pornography use were considered in an effort to test whether the prediction surface was better characterized by a “saddle” or by an inverted U shape. Finally, random effects were considered to determine if significant variation in the pornography components of the model remained unaccounted for.

With respect to relationship satisfaction, adding quadratic terms for actors’ and partners’ solitary pornography use did not significantly improve fit, χ^2^ (2) = 0.35, *p* = .859. When random effects were added for each fixed effects in the model, estimation would not converge unless the random effect for shared pornography use and the random effect for the interaction between actors’ and partners’ solitary pornography use were omitted. The addition of random effects for actors’ and partners’ solitary pornography use significantly improved model fit, χ^2^ (2) = 15.96, *p* < .002, though neither of these variance estimates were significantly different from zero.

Results were similar for sexual satisfaction. Adding quadratic terms of actors’ and partner’s solitary pornography use to the model discussed in the manuscript did not significantly improve model fit, χ^2^ = 0.211, *p* = .900. The addition of random actor and partner effects as variance components to the model, significantly improved model fit, χ^2^ (2) = 15.96, *p* < .002, although again, neither of these variance estimates were significantly different from zero.

Such results indicate that relationship and sexual satisfaction are best predicted by the saddle-shaped type of similarity-dissimilarity in solitary pornography use reported by Kohut et al. (2018) and that there are unlikely to be further couple-level moderators of the associations indicated by these models.

## Study 2

After time was tested for inclusion in the models tested in Study 2 and interaction effects were not found, quadratic effects for actors’ and partners’ attitudes towards pornography were considered. Finally, random effects were added to determine if significant variation in the pornography components of the model remained unaccounted for.

Adding quadratic attitudinal components to the model predicting relationship satisfaction without gender or time effects did not improve model fit, χ^2^ (2) = 8.97, *p* = .356. A model that considered a random intercept for couples, and random slope effects for actors’ and partners’ attitudes towards pornography would not converge unless the random slopes for actors’ and actors’ by partners’ attitudes towards pornography were removed. The resulting model improved model fit, χ^2^ (2) = 44.84, *p* < .001, and both the variance of the intercept, *Wald Z* = 4.13, *p* < .001, and the variance of the slope for partner’s attitudes towards pornography, *Wald Z* = 2.95, *p* = .003, were significant. Such results indicate that couples differed significantly in their average level of relationship satisfaction, and that the association between partner’s attitudes toward pornography and relationship satisfaction varied significantly from couple to couple which may indicate the presence of further moderating effects.

Unlike the models tested until this point, adding quadratic effects for actors’ and partners’ attitudes to the model predicting sexual satisfaction significantly changed the fit of the model, χ^2^ (2) = 13.22, *p* = .001, largely due to a significant quadratic effect for partners’ attitudes towards pornography use, *b* = 5.85, *p* = .002. In this case, RSA indicated a significant curve along the line of congruence, *a_2_* = 13.18, *p* < .001, but not along the line of incongruence, *a_4_* = -1.57, *p* = .720, as well as a significant slope along the line of incongruence, *a_3_* = 4.65, *p* = .025, but not along the line of congruence, *a_1_* = 4.17, *p* = .267. These results suggest that sexual satisfaction was highest among couple members that both strongly agreed that they enjoy pornography or strongly disagreed that they enjoy pornography, and was lower among couples who are similar in their indifference towards pornography. Importantly, unlike previous patterns of results, it appears that couples who are similarly indifferent towards pornography do not have higher sexual satisfaction than couples who are dissimilar in their attitudes towards pornography. Moreover, the slight slope along the line incongruence indicates that participants who do not enjoy pornography but have a partner that does, report somewhat higher satisfaction than participants who enjoy pornography a great deal but have a partner that does not. Because of nature of this prediction surface, which was more “U” than saddle-shaped, it seems unlikely that this operationalization of attitudes towards pornography would statistically account for similarity-dissimilarity effect of solitary pornography use identified in Study 1.

Finally, with respect to the prediction of sexual satisfaction, random effects models would not converge unless they were limited to random effects for actors’ attitudes towards pornography use and for the interaction between actors’ and partners’ attitudes towards pornography. The inclusion of these components significantly improved model fit, χ^2^ (3) = 32.70, *p* < .001 though neither of the variance estimates were significantly different from zero.

## Study 3

After gender was tested for inclusion in the models, quadratic effects for actors’ and partners’ pornography use were considered. After which, the addition of random effects were explored.

Adding quadratic terms for actors’ and partners’ solitary pornography use to the model that predicted relationship satisfaction which included a main effect for gender did not significantly improve model fit , χ^2^ (2) = 5.22, *p* = .073. The addition of random slopes for partners’ solitary pornography use significantly improved model fit, χ^2^ (1) = 11.83, *p* < .001. The variance component differed significantly from zero, *s^2^* = 0.25, *p* = .014, indicating unaccounted for variance in the slope of partners’ pornography use. Models with additional random effects for the other pornography components would not converge.

As with relationship satisfaction, adding quadratic terms of actors’ and partner’s solitary pornography use to the model predicting sexual satisfaction that contained a main effect for gender did not significantly improve the model, χ^2^ = 1.26, *p* = .534. The addition of random actor and partner effects as variance components to the model, however, significantly improved model fit, χ^2^ (2) = 14.02, *p* < .001, though neither of these variance estimates were significantly different from zero. Models with additional random effects for the other pornography components would not converge.

## Study 4

Quadratic effects for actors’ and partners’ solitary pornography use were also considered after temporal components were examined. After all fixed effects were considered, random effects were considered to determine if further unaccounted for variance in the fixed effects remained.

Adding quadratic terms for actors’ and partners’ solitary pornography use to the model predicting relationship satisfaction without gender and time components significantly improved fit, χ^2^ (2) = 6.15, *p* = .046, but neither the co-efficient for squared actors’ solitary pornography use, *b* = 0.04, *p* = .069, nor the co-efficient for the squared partners’ solitary pornography use, *b* = -0.02, *p* = .322, were significant on their own so these components were ignored. When random components were added to the model, it would not converge unless a random slope component for partners’ solitary pornography use was omitted The remaining components significantly improved fit, χ^2^ (4) = 240.56, *p* < .001, but only the random effect for actors’ solitary pornography use was significant, *s*^2^ = 0.04, *p* = .044.

Adding quadratic terms for actors’ and partners’ solitary pornography use to the model predicting sexual satisfaction without gender and time significantly improved model fit, χ^2^ (2) = 7.72, *p* = .021, but again, neither quadratic term was statistically significant. When random effects were added to the model with a main effect for time, the model would not resolve unless the random effect for the interaction between actors’ and partners’ solitary pornography use, and random slope for shared pornography use were removed. The resulting additions significantly improved model fit, χ^2^ (3) = 169.83, *p* < .001, but none of the random slope components for pornography use were significant.

Adding quadratic terms for actors’ and partners’ solitary pornography use to the model predicting sexual dissatisfaction without gender or time components did not significantly improve fit, χ^2^ (2) = 2.37, *p* = .306. When a random intercept and random slope for each pornography component were added to the model with gender and time components in the model would not resolve unless the random slope for shared pornography use was removed. The resulting additions significantly improved model fit, χ^2^ (4) = 203.39, *p* < .001, and suggested that there was significant couple-level variation in the effect of actors’ solitary pornography use, s^2^ = 0.14, *p* = .001, that remained to be accounted for.

## Discussion

The null effects for the quadratic terms in Study’s 1, 3 and 4, generally confirm the previously reported “saddle” shaped prediction surface rather than the expected inverted “U” shaped surface that is more prototypical of similarity-dissimilarity effects. The consideration of further random slope effects was not particularly informative, though it does appear that some couple-level variation in some of the models remains to be explained.
