# Supplementary material for: But What’s Your Partner Up to? Associations Between Relationship Quality and Pornography Use Depend on Contextual Patterns of Use Within the Couple
Source: Front Psychol. 2021 Jul 30;12:661347. doi: 10.3389/fpsyg.2021.661347 (PMC8362880; doi:10.3389/fpsyg.2021.661347)
Supplement: Supplementary file 2 [file Data_Sheet_2.docx]

# Supplement 2: Justification for Using Time Invariant Indicators of Pornography Use

The analytic plan that we registered for this study was premised on the use of time varying measures of pornography use and the results of these analyses can be requested from the first author. While results generally supported the anticipated associations across both relationship and sexual satisfaction, in some analyses, extent of support varied from model to model. Upon closer examination, we recognized a problem with the time varying measure of pornography use. Specifically, there appeared to be a disproportionately high number of non-using women in each wave. We believe this occurred because the measures used in this study inquired about the frequency of pornography use in the last week, despite the fact that most female pornography users report using pornography one to three times a month or less often (Carroll et al., 2008; Kohut et al., 2017a). Indeed, when women who reported some solitary pornography use in at least one wave of the study were examined more carefully, we found that the time varying measure of pornography use incorrectly characterized non-trivial proportions of these women as non-users in each wave (20%-34%, depending on the wave). The net effect of this inconsistency was to reduce the total number of observations in which both men and women within a couple reported solitary pornography use at a given wave by slightly more than half: although 37% of couples could be characterized as relationships in which both couple members used pornography alone, when examined on a wave-by-wave basis, only 18% of the total number of paired observations across waves indicated instances when both couple members reported some pornography use. Because the current hypotheses and theorizing were based on the assumption that similarity-dissimilarity effects of solitary pornography use are the result of similarity-dissimilarity of couple members, rather than the impact of pornography use per se, we decided to re-run the analyses using averaged reports of pornography use across waves to create more stable time-invariant estimates of the frequencies of pornography for each participant. Replacing time-varying reports with these time-invariant aggregates removed our ability to analyze temporal effects as we originally intended, but had the benefit of increasing our resulting sample size by 41%, from an original *n* = 197 couples to *n* = 277 couples by replacing missing values on measures of wave-specific pornography use.
